# Hematological Consequences of Environmental Change During Dewilding of Rhesus Macaques

**DOI:** 10.21203/rs.3.rs-7767375/v1

**Published:** 2025-10-16

**Authors:** Annabelle Smith, Kasalina Kiwanuka, Gabriela Pessenda, Andrew R. Rahmberg, Jacob K. Flynn, Richard Herbert, Jason M. Brenchley, P’ng Loke

**Affiliations:** 1 Type 2 Immunity Section, Laboratory of Parasitic Diseases, NIAID, NIH, Bethesda, Maryland, USA; 2 Barrier Immunity Section, Lab of Viral Diseases, NIAID, NIH, Bethesda, Maryland, USA; 3 Integrated Data Sciences Section, Research Technologies Branch, NIAID, NIH, Bethesda, Maryland, USA; 4 Comparative Medicine Branch, NIAID, NIH, Bethesda, Maryland, USA

## Abstract

The environment shapes immune system development and the regulation of inflammatory responses, which may affect the prevalence of immune mediated inflammatory diseases. However, the hematological consequences of a major environmental change, such as those experienced during migration, remain poorly understood. Here, we used cellular and molecular approaches to assess the immunological consequences in rhesus macaques as they transitioned from an outdoor provisioned environment to an indoor laboratory facility in a process we term ‘dewilding.’ Dewilding led to a sharp decrease in neutrophils and increased lymphocytes in the peripheral blood, a skewing toward a proinflammatory T_H_1 response, and increased T cell activation. Concurrently, we observed changes in the microbiome, with fungal abundance decreasing while bacterial abundance increased during dewilding. In the bone marrow, we observed increased granulopoiesis, reduced lymphocytes, and reduced hematopoietic stem cells and progenitors, with their shift toward less committed progenitor states. Single-cell nuclear RNA sequencing of the bone marrow revealed increased erythrocyte progenitors in the bone marrow during dewilding, with upregulation of genes involved in hemoglobin control and erythropoiesis. Notably, the vaccination response against measles varied based on vaccination period during the dewilding process. Together, our findings illustrate how dewilding alters immune homeostasis, with implications for understanding immune adaptation in migrants from rural to urban environments and for optimizing immunization strategies during environmental change.

## Introduction

The immune system can be shaped by environmental and lifestyle factors. Environmental drivers of immune traits include nutrition, aging, seasonal variation, and the microbiome. Migration and industrialization continue to introduce humans to environmental changes, with immunological consequences including the changing prevalence of immune mediated inflammatory diseases ^1^. The ‘Hygiene Hypothesis’^2,3^ proposed that less hygienic environments helped protect against the development of atopy, while the ‘Old Friends Hypothesis’^4^ posited that microbes that co-evolved with humans are protective against a range of inflammatory diseases. More recently, the microbiome has been characterized as a regulator of the immune response, with less diverse microbiota composition, commonly found in westernized communities, contributing to inferior immune regulation^5^. Concurrently, decreased exposure to common microbial pathogens and commensals reduces the body’s efficiency at producing immune-regulating regulatory T (T_Reg_) cells^6–8^. This inefficiency increases the risk of allergic responses to common environmental antigens and decreases the body’s defense against autoimmune disease. However, continuous exposure to antigens and persistent challenges of the immune system can lead to naïve T cell depletion, heightened immune activation, and skewing of the immune system, and increased immunological aging that may affect responses to vaccines^9^. To better understand these complex interactions, systems immunology approaches have begun to decipher the relative contributions of environmental and genetic factors to the variation in the human immune system^10–12^ .

Recently, we have performed immunological studies releasing laboratory mice into an enclosed, outdoor, enclosure, a process we call ‘re-wilding,’ to examine genetic and environmental contributions to immune variation. Rewilded mice demonstrated greater gastrointestinal (GI) microbiome diversity (both prokaryotic and eukaryotic) and expanded granulopoiesis compared to laboratory mice^13^, and they became more susceptible to infection with the intestinal nematode parasite *Trichuris muris*^14^, which dramatically influences mucosal immunity and protection against bacterial infections^15^. The re-wilding-induced increase in granulocyte populations in the blood made them more ‘human-like,’ as mouse blood usually has far fewer granulocytes than human blood. Fungal colonization was identified as a major driver of this increased granulopoieses^16^, and it conferred greater long-term protection from bloodstream infection by Gram-positive bacteria than by transient exposure to the fungal cell wall component β-glucan^13^. By flow cytometry profiling, we found that the cellular composition in both the peripheral blood and the mesenteric lymph nodes was shaped by interactions between genotype and environment^17^, which is consistent with some human studies^18,19^ and murine studies of pet shop and wild microbiome associations^20,21^. B cells also underwent major changes following rewilding, with increased signs of maturation and heightened germinal center responses within secondary lymphoid organs^22^. There were also expanded B cells in the GI tract, which was accompanied by elevated systemic levels of immunoglobulin G (IgG) and IgM antibodies reactive to the microbiota. Whereas variation in the expression of CD44 on T cells was driven mostly by genetics, expression of CD44 on B cells was explained more by the environment across different inbred strains of mice^17^. When we tracked individual mouse behavior with subcutaneous radio-frequency identification and associated it with their immune phenotype, we found that social association between mice was particularly predictive of similar memory T and B cell profiles and was more influential than sibling relationships or shared infection status^23^. Consistent with some observations in humans^24,25^, the more socially associated two individuals were, the more similar their immune phenotypes were, with this effect particularly strong for cellular composition but weaker for cytokines.

While rewilding may bring a laboratory mouse closer to the human immune system^26^, non-human primates are better for modeling human immunology, with the advantage of performing experimental interventions that are difficult in clinical studies^27,28^. Hence, we have developed a ‘de-wilding’ model transitioning rhesus macaques (*Macaca mulatta*) from a provisioned outdoor environment to indoor research facilities. Using this model, we have previously shown that transition to captivity has a large impact on the composition of the GI tract bacterial microbiome^29^. This transition mirrors human migration from rural developing regions to more industrialized urban environments, providing a unique opportunity to study the molecular and cellular effects of environmental changes on the immune system. Here, we observe some parallels with previous rewilding projects in that neutrophils are decreased and lymphocytes increased in the peripheral blood after dewilding, which is the opposite of rewilding. However, T cell activation markers are increased in dewilding, just as they are increased in rewilding. Therefore, the effects of environmental change may have distinct influences on myeloid cells compared to T cell differentiation, which led us to further examine the bone marrow in dewilded macaques to investigate hematopoietic stem and progenitor cell (HSPC) populations. Our findings provide valuable insights into how environmental changes impact the immune system, revealing that dewilding distinctly influences the differentiation trajectory of hematopoietic stem cells and subsequently affects the populations of immune cells in the peripheral blood, notably T cells and granulocytes.

## Methods

### Study design

The aims of this study were to examine how the environmental change of dewilding, the transition from a provisioned outdoor environment to indoor research facilities, affects the immune system of rhesus macaques. In study 1, 16 rhesus macaques (all male, with an average age of 2 years), were assessed before and after a 3-month quarantine in a lab environment. The macaques were cohoused in pairs to simulate social living conditions, and their diet was standardized to a controlled laboratory diet, which was consistent throughout the study period. Measurements included complete blood count (CBC) with differential of whole blood, in-vitro stimulation of whole blood and cytokine profiling with the LEGENDplex assay (BioLegend), and peripheral blood mononuclear cells (PBMC) isolation for spectral flow cytometry.

In study 2, 22 rhesus macaques (all male, with an average age of 1 year) were assessed at weeks 1, 3, 5, 7, and 9 of the dewilding quarantine period. Similarly to study 1, the macaques were cohoused in pairs, and their diet was maintained consistently with the laboratory standard. Measurements included CBC with differential of whole blood, spectral cytometry of BM, and single-nucleus RNA-sequencing of BM samples. We also evaluated the measles vaccination response in macaques during the dewilding process by randomly assigning 11 macaques into two groups: early vaccination at week 9 or late vaccination at week 23 of dewilding. Serum samples were collected five weeks following vaccination to assess the immune response.

For our analysis of the fungal and bacterial composition of fecal samples during dewilding, we assessed a third group of 17 dewilded rhesus macaques (all male). These macaques were housed consistently with the conditions of the other studies, including cohousing in pairs and maintaining a standardized laboratory diet. Fecal samples were collected at day 7 and day 18 of the dewilding process, and DNA was then extracted from the stool samples to analyze fungal and bacterial composition with qPCR.

For all analyses, samples that failed quality control, such as flow cytometry staining errors, were not included in downstream statistical analyses. The number of animals per group included in statistical analyses and the statistical tests used are reported in the figure legends. All data points are biological replicates.

### Animals

We collected data from 55 rhesus macaques for analyses. All rhesus macaques were male and aged at either 1 or 2 years. These rhesus macaques were given one dose of ivermectin (0.2 mg/kg of body weight by subcutaneous injection) following their initial exam as well as fenbendazole (50 mg/kg by oral administration) once a day for 3 days after the initial exam. Samples were taken one week after the initial exam (Week 1) and then throughout the dewilding quarantine period, as previously described.

The NIAID institutional animal care and use committee, as part of the NIH intramural research program, approved all experimental procedures pertaining to NHPs (protocol LVD 26E). The animals in this study were housed and cared for under the supervision of the Association for the Assessment and Accreditation of Laboratory Animal Care (AAALAC)-accredited Division of Veterinary Resources and as recommended by the Office of Animal Care and Use nonhuman primate management plan. Care at these facilities met the standards set forth by the Animal Welfare Act, animal welfare regulations, United States Fish and Wildlife Services regulations, as well as the 8th edition of the Guide for the Care and Use of Laboratory Animals^64^. The physical conditions of the animals were monitored daily. The animals were provided continuous access to water and offered commercial monkey biscuits twice daily as well as fresh produce, eggs and bread products, and a foraging mix consisting of raisins, nuts and rice. Enrichment to stimulate foraging and play activity was provided in the form of food puzzles, toys, cage furniture, and mirrors. All animals had the same base diet (LabDiet, St. Louis, MO, USA).

### Sample collection and CBC

Whole blood samples (approximately 30–50 μl) were collected at the timepoint of interest into EDTA-containing tubes for both spectral cytometry and complete blood count with differential (CBC). CBC was completed using the Element HT5 Veterinary Hematology Analyzer (Heska). Plasma was isolated from whole blood by centrifugation at 2,000 rpm for 10 min at 22°C. BM blood samples were aspirated from long bones into EDTA-containing tubes. For BM sample collection, rhesus macaques were sedated using tiletamine and zolazepam (Telazol) at a dosage of 3 to 4 mg/kg administered intramuscularly. Additionally, Buprenorphine Extended-Release, a compounded opioid providing up to 48 h of analgesia, was administered subcutaneously to ensure adequate pain management during and after the procedure.

Stool was collected from the bottom of individual macaques’ cages, placed inside polypropylene tubes, then flash frozen on dry ice before being stored at −80°C.

### Preparation of single-cell suspensions for PBMCs and BM

Heparinized whole blood was spun for 10 min at 1,500 rpm and plasma was collected and stored at −80 °C for further cytokine analysis. The cellular component re-suspended in PBS next underwent a density gradient separation process using the Lymphocyte Separation Media (LSM MP Biomedicals) according to the manufacturer’s instructions. Isolated PBMCs were washed twice in PBS and then used for downstream spectral cytometric analysis. PBMC isolation was performed on samples from the 16 macaques from study 1.

Single-cell suspensions were prepared from BM blood samples by thawing with water bath and warm RPMI, 2 washes with RPMI followed by centrifuging at 300× g for 7 min and lysing red blood cells in 1 mL ACK Lysis buffer (Thermo Fisher Scientific) for 2 minutes.

### Spectral Cytometry

Single-cell suspensions prepared from PBMCs or BM were washed twice with phosphate buffered saline (PBS) before incubating with Live/DeadFixable Blue (Thermo Fisher Scientific) for 20 min at 4°C. For the intracellular PBMC panel, cells were next incubated in eBioscience Transcription Factor Fixation and Permeabilization solution (Invitrogen) for 12–18h at 4 °C and stained with cocktails of fluorescently labeled antibodies against intracellular antigens (full antibody list is in Figure S3) and Fc Block (Human TruStain, BioLegend) diluted in Permeabilization Buffer (Invitrogen). For the remaining surface receptor panels, cocktails of fluorescently conjugated antibodies (full antibody list is in Figures S3 and S6) diluted in FACS Buffer (0.5g BSA, 1 mL EDTA, 500 mL PBS), Fc Block (Human TruStain, BioLegend), and 10% brilliant stain buffer (BD) were then added directly to cells and incubated for a further 30 min at 4°C. Spectral unmixing was performed for each experiment using single-strained controls using UltraComp eBeads (Invitrogen). Dead cells and doublets were excluded from analysis. All samples were collected on an Aurora spectral cytometer (Cytek) and analyzed using the OMIQ platform (https://omiq.ai/) for manual gating and clustering of different populations. Gating strategies are shown in Figures S4, S8–11.

### In vitro stimulation

A single-cell suspension of PBMCs was reconstituted in RPMI at 2 × 106 cells per milliliter, and 0.1 ml was cultured in 96-well microtiter plates that contained 10^7^ colony-forming units per milliliter of UV-killed microbes or PBS control. The stimulated microbes are as following: *B. vulgatus* (ATCC 8482), *C. albicans* (UC820), *C. perfringens* (NCTC 10240), *S. aureus* (USA300 AH-LAC), *P. aeruginosa* (PAO1), *B. subtilis* (ATCC 6633), and House Dust Mite (HDM; Stallergenes Greer) prepared in house as previously published^31,65^. Supernatants were collected after 2 days and stored at −80°C. Concentrations of IL-23, IL-12p40, CXCL10, IL-6, IL-10, IL-8, IL-1β, IFN-γ, CCL2, IFN-β, TNF-α, GM-CSF, and IL-17 in supernatants were measured using a commercially available human T helper cytokine LEGENDplex assay (Biolegend) according to the manufacturer’s instructions. Cytokine levels that were lower than the limit of detection across samples were excluded from further analysis.

### Single-nuclear RNA-seq library construction

Using the Chromium Next GEM Single Cell 3′ Kit v3.1, 4 reactions (1000269, 10X Genomics), Chromium Next GEM Single Cell 3′ Gel Bead Kit v3.1, 16 reactions (PN-1000122) and Chromium Next GEM Chip G Single Cell Kit (PN-1000120, 10X Genomics), nuclei isolated from 4 frozen BM samples (10X Protocol: Isolation of Nuclei for Single Cell RNA Sequencing & Tissues for Single Cell RNA Sequencing) were processed to generate single-cell Gel Beads-in-emulsion (GEMs) according to the manufacturer’s protocol. Approximately 2,000 nuclei per sample were loaded onto a Chromium Single Cell Controller to create GEMs. Cells were lysed, and poly(A) RNA was barcoded during reverse transcription using a Thermo Fisher Veriti 96-well thermal cycler at 53°C for 45 minutes, followed by 85°C for 5 minutes. The resulting cDNA was generated and amplified. Quality control and quantification of the cDNA were performed using the Agilent Genomic DNA ScreenTape Analysis kit (5067-5366 for Genomic DNA Reagents and 5067-5365 for Genomic DNA ScreenTape) in the TapeStation system. Single-cell RNA-seq libraries were constructed using the Chromium Next GEM Single Cell 3′ Library Kit v3.1 (PN-1000158, 10X Genomics) and sequenced on a NovaSeq Xplus platform. The sequencing run was configured as a 28:10:10:90 cycle run, targeting 10,000 cells with 25,000 reads per cell.

### Analysis of single-nuclear RNA-seq data

The analysis single-nucleus RNA-seq samples was performed with the Cell Ranger 9.0.1 software. After assessing the UMI counts vs. barcodes graphs, the number of cells captured ranged from 1,250 to 1,400 and the mean reads per cell ranged from 84,790 to 214,143. Cells with low numbers of UMI counts were filtered out. The results from Cell Ranger were processed in R 4.5.0 with the Seurat (v5.3.0) package. Quality control filtering was applied to each sample to eliminate downstream analysis of empty droplets, low-quality cells, and potential doublets. Then, we used the Seurat package to perform calculation of the number of unique genes detected in each cell (nFeature_RNA), the total number of molecules detected within a cell (nCount_RNA), and the percentage of reads that map to the mitochondrial genome. We selected the 900 cells from each sample with the highest nFeature_RNA that also contained <10% mitochondrial reads, which limited sample 3 to 706 cells, leading us to take a random sample of 706 cells from the other 3 samples. Data were normalized and scaled prior to dimensionality reduction using principal component analysis (PCA). An elbow plot was used to determine the number of principal components for downstream analysis. Unsupervised clustering was performed using a shared nearest neighbor modularity optimization algorithm, followed by non-linear dimensionality reduction with UMAP for visualization. Marker genes for each cluster were identified using Wilcoxon rank-sum tests and then utilized to annotate clusters manually. Differentially expressed genes (DEGs) between dewilding timepoints were computed using the FindMarkers function with multiple thresholds. Visualization included PCA and UMAP plots, violin plots, dot plots, and heatmaps of top marker genes. All figures and tables were generated in R (v4.5.0).

### Determination of fungal and bacterial quantity during dewilding by qPCR

DNA was isolated from fecal samples with bead beating followed by Qiagen Symphony DNA extraction, as previously described^66^. We then performed quantitative PCR for fungus with the FungiQuant assay and for bacteria with a 16S assay. We used the TaqMan^™^ Fast Advanced Master Mix for qPCR (Thermo Fisher Scientific cat. 4444963). We tested 2 μL of DNA at a concentration of 0.1 ng/μL. The sequence of the forward primer for FungiQuant was 5′-GGRAAACTCACCAGGTCCAG-3′, the reverse primer sequence was 5′-GSWCTATCCCCAKCACGA-3′, and the probe sequence was 5′-TGGTGCATGGCCGTT-3′. For the 16S assay, the sequence of the forward primer was 5’-CGGTGAATACGTTCYCGG -3’, the reverse primer sequence was 5’- GGWTACCTTGTTACGACTT -3’, and the probe sequence was 5’-CTTGTACACACCGCCCGTC-3’. The standard curve for each PCR run was generated using FRODO 16S standards^30^ to calculate the bacterial copy number. Reaction conditions were the following: 50°C for 2 min, 95°C for 2 min, and 45 cycles of 95°C for 1 s, followed by 60°C for 20s. We used the QuantStudio^™^ 3 System PCR machine (Thermo Fisher Scientific), and we performed the analysis with QuantStudio^™^ Design and Analysis Software (Thermo Fisher Scientific).

### Determination of measles titers by ELISA

Measles titers from serum samples were quantified using a commercially available Human Measles Virus IgG (MV IgG) enzyme-linked immunosorbent assay (ELISA) kit (Abbexa). Samples were assayed at a 101-fold dilution per the manufacturer’s protocol. Relative Measles IgG concentrations pre and post-vaccination were calculated based on optical density readings.

### Statistical Analyses

Statistical analyses were conducted using Prism version 10 (GraphPad Software Inc.) Statistical parameters including the exact number (N) of rhesus macaques are annotated in the corresponding figure legend. Data presented in bar plots display the mean. Assumptions of normality and homogeneous variance were assessed to guide selection of statistical tests. Differences between dewilding timepoints were assessed using paired two-tailed t tests, unless otherwise indicated. Numerical *P* values are indicated in the figures, with *P* values ≤0.05 considered significant.

### Visualization

Data were visualized using R studio (v4.5.0), GraphPad Prism software (v10.5.0), and Seurat (Seurat v5.3.0; scRNA-seq data). Cartoon graphics were created using BioRender.com.

#### Data Availability

RNA-seq data were deposited into the Gene Expression Omnibus database under accession number GSE301748.

## Results

### Dewilding decreases peripheral granulocytes and increases peripheral lymphocytes

**Figure 1.**
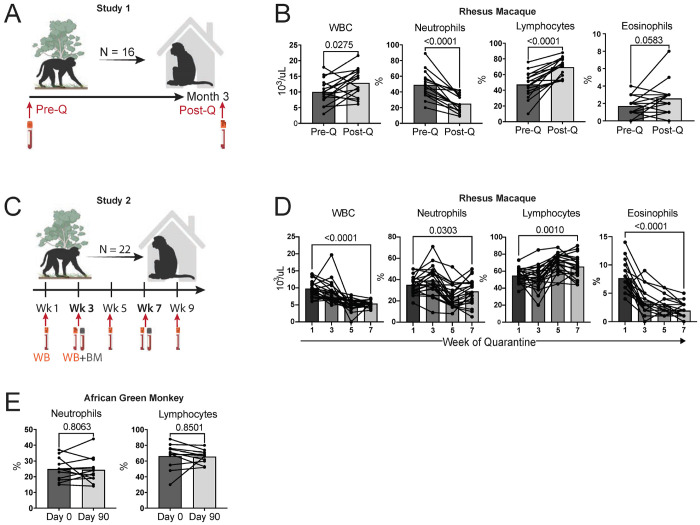
Dewilding leads to reduced neutrophils and increased lymphocytes. (A). Schematic of dewilding study 1 (N=16) showing whole blood (WB) sampling of rhesus macaques for CBC (Fig. 1), in vitro stimulation (Fig. 2), and flow cytometry (Fig. 3) before (Pre-Q) and after 3-month quarantine in a lab environment (Post-Q). (B) Before-and-after bar plots showing the percentages of white blood cells (WBC), neutrophils, lymphocytes, and eosinophils measured in rhesus macaques before (Pre-Q) and after 3 months of quarantine in a lab environment (Post-Q), as assessed by complete blood count (CBC) with differential (N=16). (C) Schematic of dewilding study 2 (N=22) showing whole blood (WB) and bone marrow (BM) sampling of rhesus macaques for CBC (Fig. 1) and flow cytometry (Fig. 3) at weeks 1, 3, 5, 7, and 9 of the dewilding quarantine period. (D) Bar plots showing the concentration of WBC, neutrophils, lymphocytes, and eosinophils measured in rhesus macaques at weeks 1, 3, 5, and 7 of quarantine in a lab environment (Post-Q), as assessed by complete blood count (CBC) with differential (N=22). (E) Before-and-after bar plots showing the percentages of neutrophils and lymphocytes measured in African green monkeys at day 0 and day 90 in a lab environment, as assessed by complete blood count (CBC) with differential. Points in bar plots represent individual rhesus macaques or African green monkeys. *P* values were determined by paired two-tailed t-test.

To determine the impact of ‘dewilding’ —the transition from an outdoor environment to a controlled quarantine environment—on the immune system, we first analyzed blood samples from rhesus macaques (N=16) before and after they completed a 3-month quarantine period after moving into an indoor facility (Figure 1A). Complete blood counts (CBC) with differential analysis showed a significant reduction of neutrophils and expansion of lymphocytes in the peripheral blood (Figure 1B). A second study (N=22) was then conducted, whereby blood was collected at several time points during the dewilding period (Figure 1C). Consistent with the first study, there was a significant reduction of neutrophils and increase of lymphocytes in the peripheral blood. However, in contrast to study 1, whereby dewilding slightly increased white blood cells (WBC) and eosinophils in the blood, in study 2 there was a sharp drop in WBCs and eosinophils during the dewilding period (Figure 1D). Other hematological parameters were not consistent or significantly altered across both the studies, including data on monocyte percentage, hematocrit, hemoglobin concentration, or red blood cell count (Figure S1). To determine if changes in neutrophils and lymphocytes were due to aging or the effects of extended time in the quarantine environment, we analyzed CBC with differential data of age-matched African green monkeys (AGMs), which never undergo environmental change (Figure 1E). AGMs did not exhibit any discernable changes in neutrophil or lymphocyte populations during a similar time. Overall, these results indicate that there are consistent alterations in the peripheral blood neutrophil and lymphocyte compartments in dewilded macaques, whereas changes in total WBCs and eosinophils varied between the two experiments (potentially due to decreased parasite burden following a deworming procedure during the transition^29^.

### Dewilding shifts a T_H_17 response to a T_H_1 response and increases activation and maturation of T cells.

**Figure 2.**
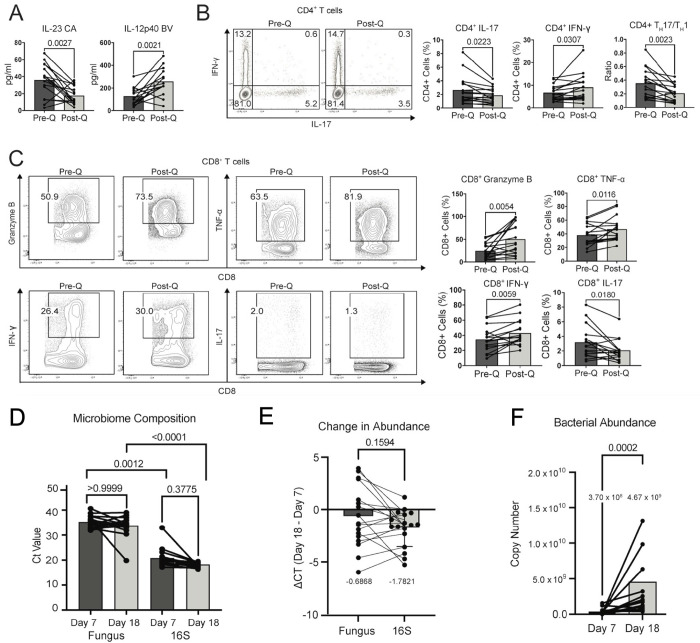
Dewilding-driven shifts in pathogen-specific immune responses and T-cell cytokine production. (A) Before-and-after bar plots showing the cytokine response to in vitro stimulation by *Candida albicans* (T_H_17) or *Bacteroides vulgatus* (T_H_1), as demonstrated by the concentration of IL-23 or IL-12p40, respectively, before and after quarantine (N=16). (B) Representative flow cytometry analysis and quantification of intracellular cytokine staining for IFN- γ and IL-17 in CD4^+^ T cells, including quantification of CD4^+^ T_H_17/T_H_1 response ratio, before and after quarantine (N=16). (C) Representative flow cytometry analysis and quantification of intracellular cytokine staining for Granzyme B, TNF-α, IFN- γ, and IL-17 in CD8^+^ T cells, before and after quarantine. (D) Before-and-after bar plots of Cycle Threshold (Ct) values from quantitative PCR (qPCR) measuring abundance of fungus and bacteria at day 7 and day 18 of dewilding (N=17). Statistical analyses shown are Dunn’s multiple comparisons test. (E) Before-and-after bar plots showing the change in abundance of fungus and bacteria in macaque stool during dewilding, calculated as the ΔCt = Ct_day18_- Ct_day7_ (N=17). (F) Before-and-after bar plots showing the bacterial copy number in macaque stool at day 7 and day 18 of dewilding (N=17). Points in bar plots represent individual rhesus macaques. *P* values were determined by paired two-tailed t-test unless otherwise stated.

To investigate how dewilding affects cytokine responses of immune cells in the blood we stimulated the whole blood of animals from study 1with different antigens (*Staphylococcus aureus* (SA), *Pseudomonas aeruginosa* (PA), *Bacillus subtilis* (BS), House Dust Mite (HDM), *Clostridium perfringens* (CP), *Bacteroides vulgatus* (BV) and *Candida albicans* (CA)) and profiled the production of cytokines (IL-23, IL-12p40, CXCL10, IL-6, IL-10, IL-8, IL-1β, IFN-γ, CCL2, IFN-β, TNF-α, GM-CSF, and IL-17) using a multiplex cytokine array. Notably, after dewilding, the IL-23 response to CA was significantly reduced and the IL-12p40 response to BV was significantly increased (Figure 2A). Additionally, other Type 1-associated cytokines CXCL10, TNF-α, and IFN-γ were increased following dewilding, but Type 2-associated cytokines were not altered by dewilding (Figure S2). These results suggest a shift from a T_H_17 response to a T_H_1 response after dewilding, which we examined by intracellular cytokine staining of peripheral blood mononuclear cells (PBMCs) (Figures S3–4). In CD4^+^ T cells, there was a significant reduction of IL-17^+^ cells and a significant increase of IFN-γ^+^ cells after dewilding (Figure 2B). Thus, the ratio of CD4^+^ T_H_17/T_H_1 cells is decreased after dewilding (Figure 2B). For CD8^+^ T cells, following dewilding, there were also significantly more Granzyme B, TNF-α, and IFN-γ positive CD8^+^ T cells (Figure 2C). While the numbers are very low, there were fewer IL-17 positive CD8^+^ T cells after dewilding (Figure 2C). Foxp3^+^ T_Reg_ populations were unaltered by dewilding (Figure S5). Apart from a slight reduction in IL-5^+^ CD4^+^ and CD8^+^ T cells and in IL-2^+^ CD8^+^ T cells, there were no other changes to cytokine positive T cells following dewilding (Figure S5). Overall, these results indicate a dewilding-induced shift from a Type 17 response to a Type 1 response, which is consistent with the reduction in neutrophil levels in the blood.

To determine if fungal and bacterial abundances in stool samples were altered during dewilding, we analyzed the DNA extracted from stool samples collected longitudinally during the dewilding process. Quantitative PCR (qPCR) analysis of fungal DNA and bacterial DNA was performed to calculate fungal and bacterial Cycle Threshold (Ct) values and bacterial copy number^30^. We calculated difference in Ct values (ΔCt = Ct_day18_- Ct_day7_) for fungal and bacterial DNA to determine changes in abundance during dewilding. As expected, fungal DNA quantities were consistently lower than bacterial DNA across all samples (Figure 2D). Notably, during dewilding, there is increasing abundance of bacterial copy numbers (Figure 2E), whereas fungal abundance remains the same. Bacterial copy number significantly increased, more than 12-fold during dewilding (Figure 2F); hence while fungal DNA levels remained relatively stable, bacterial density in the stool increased during dewilding. The decline in the relative fungal:bacterial ratio during dewilding might play a role in the shift from a Type 17 response to a Type 1 response.

**Figure 3.**
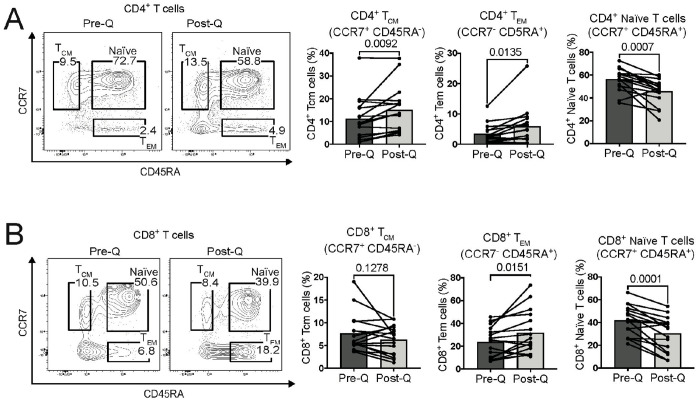
Dewilding-driven shifts in T-cell populations. (A-B) Representative flow cytometry analysis and quantification of central memory (T_CM_), effector memory (T_EM_), and naïve T cells for CD4^+^ (A) and CD8^+^ (B) T cells, before and after quarantine (N=16). Points in bar plots represent individual rhesus macaques. *P* values were determined by paired two-tailed t-test.

We previously found that during rewilding, mice increase T effector memory (T_EM_) and T central memory (T_CM_) cells while reducing naïve T cells in the blood^16,31^. Here, we find that dewilding macaques also increases CD4^+^ effector and central memory T cells, while naïve T cells were reduced after dewilding (Figure 3A). CD8^+^ effector memory T cells were also increased during dewilding, and naïve T cells were reduced following dewilding (Figure 3B). However, no significant changes were observed in the population of CD8^+^ central memory T cells. Consequently, while some aspects of dewilding are consistent with reversing the effects of rewilding (i.e. neutrophil populations in the blood), other immune features of rewilding (i.e. T cell maturation/activation) were not altered by dewilding, indicating that both types of environmental changes mature T cell populations in the blood overall.

### Dewilding induces distinct changes to hematopoietic precursor cells in the bone marrow

**Figure 4.**
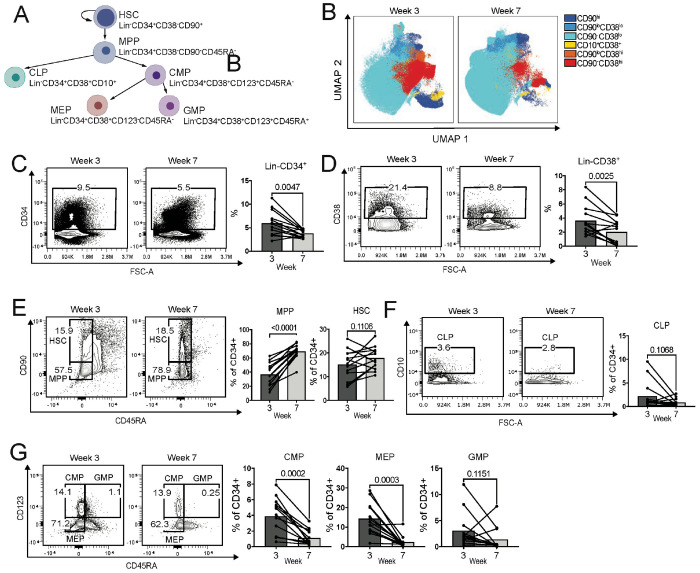
Dewilding-driven shifts in hematopoietic compartment and progenitors in the bone marrow. (A) Schematic of hematopoietic stem and progenitor cell populations and their distinctive cellular markers to be assessed by flow cytometry. (B) UMAP visualization showing the changes in hematopoietic stem cell (HSC) populations based on CD90 and CD38 expression, derived from flow cytometry analysis of bone marrow cells from dewilded rhesus macaques, observed at week 3 and week 7 of dewilding (N=13). (C) Representative flow cytometry analysis and quantification of CD34^+^ hematopoietic stem cell compartment at week 3 and week 7 of dewilding (N=13). (D) Representative flow cytometry analysis and quantification of CD38^+^ hematopoietic progenitor cell compartment at week 3 and week 7 of dewilding (N=13). (E) Representative flow cytometry analysis and quantification of CD34^+^CD38^−^CD90^+^CD45RA^−^ hematopoietic stem cells (HSC) and CD34^+^CD38^−^CD90^−^CD45RA^−^ multipotent progenitor (MPP) cells at week 3 and week 7 of dewilding (N=13). (F) Representative flow cytometry analysis and quantification of CD34^+^CD38^+^CD10^+^ common lymphoid progenitor (CLP) cells at week 3 and week 7 of dewilding (N=13). (G) Representative flow cytometry analysis and quantification of Lin^−^CD34^+^CD38^+^CD123^+^CD45RA^−^ common myeloid progenitor (CMP), Lin^−^CD34^+^CD38^+^CD123^−^CD45RA^−^ megakaryocyte-erythroid progenitor (MEP), and Lineage ^−^CD34^+^CD38^+^CD123^+^CD45RA^+^ granulocyte-monocyte progenitor (GMP), at week 3 and week 7 of dewilding (N=13). Points in bar plots represent individual rhesus macaques. *P* values were determined by paired two-tailed t-test.

Rewilding laboratory mice can enhance granulopoiesis in the bone marrow (BM)^13^. Thus, we collected BM samples from the macaques during the dewilding period (Figure 1C) for flow cytometry analysis (Figures S6–7). We focused our analysis on samples collected in the first and second month of dewilding, in weeks 3 and 7, where major changes in the blood were observed by CBC with differential analysis (Figure 1D). We first investigated hematopoietic stem and progenitor cells (HSPCs) by analyzing CD34^+^ cells along with several differentiation and progenitor markers (CD10, CD38, CD45RA, CD90, CD123; Figure 4A, Figures S6A, S7–8).

Dimensionality reduction of the (13-color panel; Figure S6A) flow cytometry data comparing week 3 to week 7 of dewilding revealed a reduction in the progenitor marker CD38 and in hematopoietic stem cell (HSC) marker CD90 expression within the CD34^+^ hematopoietic compartment (Figure 4B). The frequency of lineage negative CD34^+^ and CD38^+^ cells are reduced during dewilding (Figures 4C–4D). Within the CD34^+^ hematopoietic compartment, the percentage of multipotent progenitor (MPP) cells was significantly increased (Figure 4E). Although common lymphoid progenitor (CLP) and granulocyte-monocyte progenitor (GMP) cells were not significantly reduced (Figure 4F–G), we observed a significant reduction in the megakaryocyte-erythrocyte progenitor (MEP) and common myeloid progenitor (CMP) (Figure 4G). Overall, there is a shift from more committed progenitors, such as the CMP cells, towards the less committed MPP cells.

**Figure 5.**
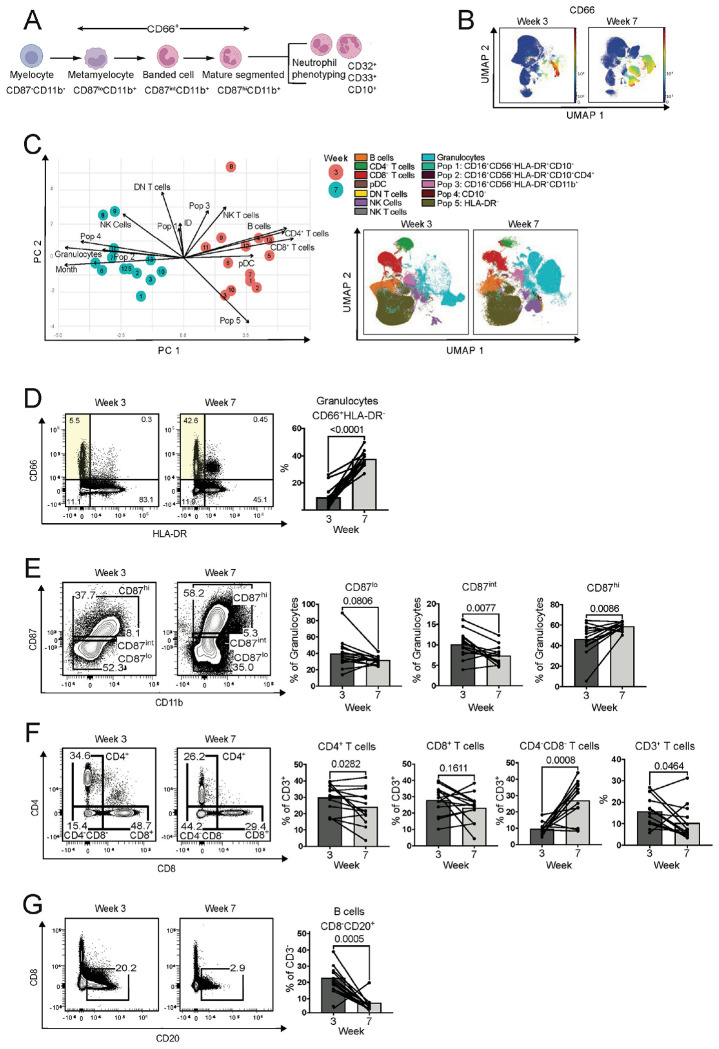
Dewilding-driven shifts in granulocytes and lymphocytes in the bone marrow. (A) Schematic of granulocyte progenitor and phenotypic populations and their distinctive cellular markers to be assessed by flow cytometry. (B) Uniform Manifold Approximation and Projection (UMAP) visualization of the change in CD66 expression in flow cytometry data from bone marrow cells of dewilded rhesus macaques, at week 3 and week 7 of dewilding (N=13). (C) Principal component analysis (PCA) and UMAP visualization of immune cell populations identified with flow cytometry data from bone marrow cells of dewilded rhesus macaques, at week 3 and week 7 of dewilding (N=13). (D) Representative flow cytometry analysis and quantification of CD66^+^HLA-DR^−^ granulocytes, at week 3 and week 7 of dewilding (N=13). (E) Representative flow cytometry analysis and quantification of neutrophil maturation, quantifying CD87^low^CD11b^+^ metamyelocytes, CD87^int^CD11b^+^ banded neutrophils, and CD87^hi^CD11b^+^ mature segmented neutrophils, at week 3 and week 7 of dewilding (N=13). (F) Representative flow cytometry analysis and quantification of CD8^−^CD20^+^ B cells, at week 3 and week 7 of dewilding (N=13). Points in bar plots represent individual rhesus macaques. *P* values were determined by paired two-tailed t-test.

We next investigated the CD34^−^ population, focusing on neutrophil maturity markers and phenotypes in the BM (Figure 5A, Figures S6B, S9–11). Uniform Manifold Approximation and Projection (UMAP) dimensionality reduction of (18-color panel, Figure S6B) flow cytometry data revealed an increase in the frequency of CD66^+^ granulocytes within the CD34^−^ population (Figure 5B), which contrasts with reduced neutrophil abundance observed in the blood. Principal component analysis (PCA) of the flow cytometry data (Figure 5C) clearly demonstrated separation of the week 3 and week 7 BM samples. PCA and UMAP analyses established that granulocytes, CD4^+^ T-cell, CD8^+^ T-cell, and B-cell populations contributed greatly to this variation between week 3 and week 7 BM samples during dewilding. UMAP dimensionality reduction confirmed that after dewilding, there were dramatically higher numbers of granulocytes, while the CD4^+^ T-cell, CD8+ T-cell, and B-cell populations were reduced after dewilding (Figure S6B).

We next confirmed by manual gating that there was a substantial increase in CD66^+^HLA-DR^−^ granulocytes in the BM during dewilding (Figure 5D). In phenotyping this granulocyte population during dewilding, the CD87^int^CD11b^+^ banded cells were reduced, while the CD87^hi^CD11b^+^ mature segmented neutrophils were increased (Figure 5E). CD32 expression was significantly reduced and CD10 expression was significantly increased in granulocytes during dewilding (Figure S12A, 12D). We did not observe distinct changes in the other neutrophil markers we assessed. (Figure S12B–C). In contrast, lymphocyte populations in the BM were reduced during dewilding (Figure 5F–G). Specifically, the overall CD3^+^ T-cell compartment, CD4^+^ T cells, and B cells were significantly reduced in the BM. CD8^+^ T cells were also reduced during dewilding, although this reduction was not statistically significant. However, double-negative T cells were notably increased during dewilding. We also observed significant changes in other immune cell populations. Following dewilding, there was an increase in the mDC to pDC ratio (Figure S12E) and reductions in non-classical monocytes, natural killer (NK) cells, and NK T cells (Figure S12F–I).

Overall, the BM results indicate that cellular changes in the blood observed during dewilding are not directly reflected in changes to the composition of the BM (Figure S13). More complex trafficking and retention mechanisms are at play contributing to changes in the hematological cells in the peripheral blood and BM during dewilding.

### Dewilding induces increased erythropoiesis

**Figure 6.**
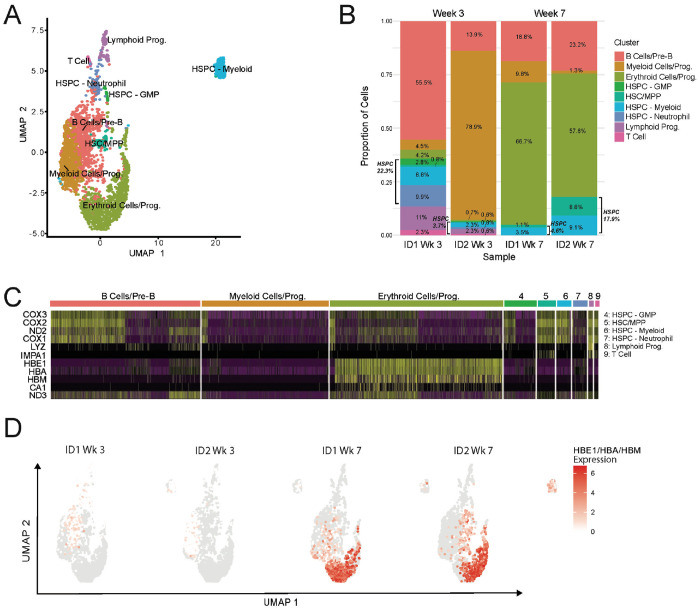
Dewilding-driven changes in cell populations and gene expression identified by single-cell nuclear RNA-Seq in the bone marrow. (A) Uniform Manifold Approximation and Projection (UMAP) visualization of the cell populations in the bone marrow identified by single-cell nuclear RNA-Seq, pooling samples from two representative animals at weeks 3 and 7 of dewilding (N=4). (B) Composition plot of bone marrow cell populations identified by single-cell nuclear RNA-Seq, showing relative abundance changes at week 3 and week 7 of dewilding (N=4). (C) Heatmap of differentially expressed genes across cell populations identified by single-cell nuclear RNA-Seq in bone marrow, highlighting distinct gene expression profiles primarily enriched in B Cells/Pre-B cells and erythrocytes (N=4). (D) UMAP visualization of the expression of hemoglobin-associated genes HBE1, HBA, and HBM in the bone marrow at week 3 and week 7 of dewilding (N=4).

To further investigate the transcriptional profile and composition of BM cells during dewilding, we analyzed single-cell nuclear RNA-Seq data from BM samples collected at weeks 3 and 7 (Figure 1C). We identified nine distinct cell clusters and characterized their respective immune cell populations (Figure 6A). The identified immune cell populations included: T Cells, Lymphoid Progenitors, Myeloid Hematopoietic Stem and Progenitor Cells, Neutrophil HSPC, Granulocyte Monocyte Progenitor (GMP) HSPC, B Cells/Pre-B, Hematopoietic Stem Cell (HSC)/Multipotent Progenitor (MPP), Myeloid Cells/Progenitors, and Erythroid Cells/Progenitors. Quantifying the changes in cluster proportions revealed a significant increase in erythroid cells and progenitors during dewilding (Figure 6B). In Animal ID1, we observed a concurrent reduction in B cells, while in Animal ID2, there was a reduction in myeloid cells and progenitors. No distinct changes were observed in the proportions of hematopoietic stem and progenitor cells. Although their proportions were low, lymphoid progenitors and T cells were reduced during dewilding.

We assessed differentially expressed genes within each cluster and noted significant changes in genes related to cyclooxygenase enzymes (COX1-3) and hemoglobin regulation (HBA, HBE1, HBM) (Figure 6C). Cyclooxygenase enzymes were particularly enriched in the following clusters: B Cells/Pre-B, HSC/MPP, HSPC - Myeloid, Lymphoid Progenitors, and T Cells. Genes associated with hemoglobin regulation were enriched in erythroid cells and progenitors, with their expression significantly increasing during dewilding (Figure 6D). Overall, our results highlight distinct changes to BM immune cell populations during dewilding, notably an increase in erythroid cells and progenitors, indicating increased erythropoiesis during dewilding.

### Dewilding influences vaccine responses

**Figure 7.**
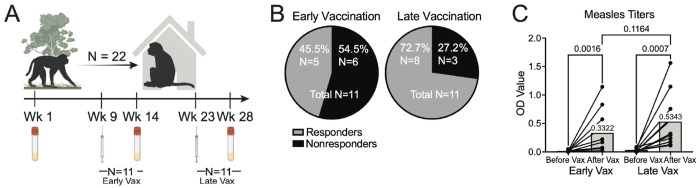
Changes to measles vaccine response given timing of vaccine during dewilding. (A) Schematic of the vaccination and serum sampling schedule, illustrating the differentiation between early (N=11) and late (N=11) vaccination groups based on the timing of vaccination during the dewilding process. (B) Pie chart showing the percentage of responders and nonresponders to measles vaccination within the early (N=11) and late (N=11) vaccination groups. (C) Before-and-after bar plots of optical density values from enzyme-linked immunosorbent assay (ELISA) measurements of measles titers pre- and post-vaccination in early (N=11) and late (N=11) vaccination groups. Statistical analyses shown are Kruskal-Wallis tests for pre- and post-vaccination comparisons within each group and Mann-Whitney test for post-vaccination comparison between early and late vaccination groups. *P* values were determined by Kruskal-Wallis test for comparisons within the vaccination groups and by two-tailed Mann-Whitney test for comparing the ‘After Vax’ timepoint between early and late vaccination groups.

There is considerable geographic variation in vaccine responses, which could be driven by multiple environmental factors^9^. Certain vaccines have high efficacy in high-income countries, whereas in low-income regions these same vaccines may achieve only 20–50% protection. To investigate if vaccine responses during dewilding is altered, measles vaccinations were administered to two groups of rhesus macaques at different timepoints during dewilding, with serum samples collected five weeks post-vaccination (Figure 7A). We performed enzyme-linked immunosorbent assays (ELISA) on serum samples before and after vaccination to evaluate measles titers in both groups. Vaccine response was defined as a conversion from negative to positive titer measurement, while nonresponse was defined by maintaining a negative titer measurement post-vaccination.

Strikingly, among the early vaccination group, there was an approximately equal distribution of responders (N = 5) and non-responders (N = 6), whereas the late vaccination group showed a majority of macaques responding to the vaccine (N = 8) (Figure 7B). The early vaccination group had twice as many non-responders (N = 6) compared to the late vaccination group (N = 3). Although the post-vaccination OD value was higher in the late vaccination group (0.5343) compared to the early vaccination group (0.3322), this difference was not statistically significant (p = 0.1164). The late vaccination group experienced a more significant increase in OD values following vaccination (p = 0.0007), compared to the early vaccination group (p = 0.0016) (Figure 7C). Overall, these results suggest the possibility that vaccination responses may improve as the individuals undergo dewilding.

## Discussion

Understanding the effects of environmental transitions on the immune system could provide significant insights into the basis of immune variation between individuals and lead to novel therapeutics aimed to improve immunological diseases. Rewilding laboratory mice into natural outdoor enclosures has demonstrated distinct alterations to immune phenotypes from environmental change, including expanded granulopoiesis and circulation of peripheral blood neutrophils, bringing laboratory mice closer to human-like immune characteristics^13,16^. Moreover, creating inbred mice that have microbiomes of wild mice leads to enhanced survival after viral infection^32^. Hence, environmental factors can have significant influence on immunity. Here, we have studied the reverse transition, employing a ‘dewilding’ model, where we transitioned rhesus macaques from a provisioned outdoor setting to a controlled indoor environment. In direct contrast to the rewilding studies, we found a significant reduction in neutrophils and an expansion of lymphocytes in the peripheral blood following dewilding. In humans, intestinal fungal colonization by *C. albicans* is associated with increased differentiation of T_H_17 CD4^+^ T cells^33^, and our previous studies on the microbiota of rewilded mice showed a significant increase in fungal colonization. Thus, we identified changes opposite to those seen during rewilding, including a shift from T_H_17 response toward a T_H_1 response and a decreased fungi-to-bacteria ratio in fecal contents, indicating a relative increase in bacterial abundance. However, we also found an increase in central and effector T cells, alongside a decrease in naïve T cell frequencies during dewilding, which mirrors the same trajectory from rewilding studies^16^. This parallelled observation indicates that both models of environmental change stimulate maturation of the T cell compartment, likely due to altered antigenic exposure and immune system activation. Hence, we find that while some aspects of dewilding appears to reverse the immunological effects of rewilding, other immune phenotypes instead follow the same trend of increased maturation with rewilding.

In this study we had the rare opportunity to characterize changes to the hemopoietic cells in the BM during a major environmental change. By flow cytometry analysis, we found significant changes to granulocyte and lymphocyte populations during dewilding. However, the changes observed in peripheral blood during dewilding were not mirrored in the BM. In peripheral blood, dewilding led to reduced neutrophils and increased lymphocytes, whereas in the BM, we found increased granulocytes and reduced lymphocytes. These contrasting observations may be attributed to dewilding-induced alterations in the dynamics of immune cell egress from the BM. Inflammatory signals can promote lymphocyte egress from the BM while retaining granulocytes, regulated by the CXCL12/CXCR4 axis and driven by cytokines like TNF-α^34,35^. During dewilding, we observed a shift towards a proinflammatory T_H_1 response, producing proinflammatory cytokines TNF-α and IFN-γ. Hence, if inflammation signals reduced lymphocyte numbers in the BM through egress, granulocyte precursors could expand into the vacated space and undergo maturation. This dynamic would be consistent with our observations of increased expression of neutrophil maturation markers, such as CD11b and CD10^36,37^, in BM granulocytes, alongside accumulating CD87^hi^ mature neutrophils during dewilding. Alternatively, the discrepancy between peripheral blood and BM findings could be explained by increased trafficking of granulocytes from the peripheral blood into tissues. This would account for the reduced neutrophil count in peripheral blood, despite evidence of increased granulopoiesis in the BM. The depletion of neutrophil reserves could trigger a feedback mechanism that enhances granulopoiesis, potentially resembling a process known as ‘emergency granulopoiesis’^38,39^.

During dewilding, the most notable change to hematopoietic stem and progenitor cell (HSPC) populations was a reduction in the overall HSPC compartment, identified by CD34^+^ cells, and in the more committed progenitors, defined as CD38^+^ cells. We found a shift from more differentiated progenitors, such as the common myeloid progenitors (CMP), towards the less committed multipotent progenitor (MPP) cells. These changes align with previously described regulation of hematopoietic stem cells (HSCs) during inflammation^40–42^, particularly influenced by TNF-α, which was elevated during the proinflammatory T_H_1 response observed in our study. Although TNF-α was traditionally understood to inhibit growth and induce apoptosis in HSCs^43,44^, recent studies have shown that TNF-α promotes survival of more primitive HSC subpopulations while inducing apoptosis in more committed progenitors^45,46^. This leads to an increased proportion of MPP cells in the BM, consistent with our findings during dewilding.

We also examined changes in the BM during dewilding at the single-cell transcriptional level and found a striking increase in erythroid cells and progenitors. Notably, this increase was not reflected in peripheral blood parameters: we did not observe significant increases in red blood cell counts, hematocrit, or hemoglobin levels during dewilding. However, the expression of genes related to hemoglobin control, including HBA, HBE1, and HBM, were significantly increased during dewilding, and their expression was localized to the expanded erythroid population, indicating that erythropoiesis had increased during dewilding. Previous studies have demonstrated that erythropoiesis induces the egress of lymphocytes from the BM to the periphery and may reduce HSCs in the BM^47,48^. One hypothesis is that there is increased interplay between inflammation and erythropoiesis during dewilding, particularly mediated by TNF-α, which could be a driver of the immunological changes observed during dewilding. This bidirectional relationship has been previously described: inflammation can trigger stress erythropoiesis that compensates for reduced steady-state erythropoiesis^49,50^, while erythropoietin can then resolve inflammation by suppressing NFκB signaling^51^. One possibility is that this inflammation–erythropoiesis axis^52^, whereby inflammation promotes erythropoiesis, and erythropoiesis contributes to resolving inflammation, may explain the shifts we observed in BM composition during dewilding. This dynamic may be further moderated by reactive oxygen species (ROS), which are elevated when HSC metabolism is reprogrammed from anaerobic glycolysis to oxidative respiration during inflammation^53,54^. Inflammation also contributes to elevated ROS levels in the BM by dampening the CXCR4/CXCL12 axis^55^ and by stimulating ROS production by BM-resident myeloid cells^56^. Notably, ROS production has been described as required for reactive granulopoiesis in response to sterile inflammatory stimuli^56^, supporting the inflammation–erythropoiesis axis as a driver of the immunological changes observed during dewilding.

Because vaccines are frequently less effective in populations from developing countries, where microbial and antigen exposure is increased compared to more industrialized areas^57,58^, we sought to investigate how dewilding influences vaccine responsiveness. Although our sample size was limited, we found that earlier vaccination relative to the onset of dewilding was associated with nonresponse to measles vaccination. In contrast, macaques vaccinated later in the dewilding process were more likely to respond to the measles vaccine and exhibited higher antibody levels post-vaccination. These findings suggest that the timing of vaccination during the environmental transition of dewilding may shape vaccine responsiveness. This insight may help explain the heterogeneity in vaccine response observed in developing regions and could inform guidelines on optimal vaccination timing for migrants transitioning to new environments.

One possibility for the heightened T_H_1 response observed during dewilding may be infection with rhesus cytomegalovirus (RhCMV), common in captive rhesus macaques^59^. Like humans, rhesus macaques typically exhibit overt CMV clinical symptoms only under immunosuppressive conditions^60,61^, but CMV infection drives large T cell clonal expansions^62,63^. It is also difficult to disentangle the individual components of the dewilding environment– such as altered diet, spatial confinement, and changes in antigen exposure– and their effects on the immune system. Nevertheless, this complexity mirrors the multifaceted nature of human migration from rural developing communities to more industrialized urban environments, where multiple environmental changes also occur simultaneously.

In summary, our results indicate that dewilding in rhesus macaques reverses some of the hematological effects of rewilding, with reduced peripheral blood neutrophils and increased peripheral lymphocytes. However, dewilding continues to mature the T cell compartment, increasing the proportion of memory T cells and reducing naïve T cells, coinciding with a shift to a T_H_1 response from a more T_H_17 response. We also documented changes to the BM during dewilding, including increased granulopoiesis, and changes in hematopoietic stem and progenitor cell populations. These shifts were accompanied by increased erythropoiesis, suggesting a dynamic interplay between immunological changes and erythropoiesis during dewilding. Notably, we also found that the timing of vaccination during dewilding could potentially influence successful vaccine responsiveness. Earlier vaccination was associated with more seroconversion failure, and these results are consistent with observations that vaccines can have lower protection in low-income regions of the world. Overall, this study characterized some of the profound impacts of environmental change on the immune system. By describing the immunological consequences of transitioning to a more sterile environment in this rhesus macaque dewilding model, future studies may eventually inform important considerations for optimizing immune interventions, such as vaccination, in human migrants undergoing environmental transition.

## Supplementary Material

1

## Key Points:

1. Environmental transition from an outdoor to a quarantined environment alters the immune landscape of the peripheral blood and bone marrow.
2. Vaccination responses and changes in fungal bacterial abundances could be associated with environmental effects.

## References

[1] Agrawal M, Shah S, Patel A, Pinotti R, Colombel JF, Burisch J. Changing epidemiology of immune-mediated inflammatory diseases in immigrants: A systematic review of population-based studies. J Autoimmun. 2019;105:102303. 31351784 10.1016/j.jaut.2019.07.002 PMC7382899

[2] Strachan DP. Hay fever, hygiene, and household size. BMJ. 1989;299(6710):1259–1260. 2513902 10.1136/bmj.299.6710.1259 PMC1838109

[3] Murdaca G, Greco M, Borro M, Gangemi S. Hygiene hypothesis and autoimmune diseases: A narrative review of clinical evidences and mechanisms. Autoimmun Rev. 2021;20(7):102845. 33971339 10.1016/j.autrev.2021.102845

[4] Rook GA, Brunet LR. Old friends for breakfast. Clin Exp Allergy. 2005;35(7):841–842. 16008666 10.1111/j.1365-2222.2005.02112.x

[5] Noverr MC, Huffnagle GB. The ‘microflora hypothesis’ of allergic diseases. Clin Exp Allergy. 2005;35(12):1511–1520. 16393316 10.1111/j.1365-2222.2005.02379.x

[6] Belkaid Y, Harrison OJ. Homeostatic Immunity and the Microbiota. Immunity. 2017;46(4):562–576. 28423337 10.1016/j.immuni.2017.04.008 PMC5604871

[7] Renz H, Skevaki C. Early life microbial exposures and allergy risks: opportunities for prevention. Nat Rev Immunol. 2021;21(3):177–191. 32918062 10.1038/s41577-020-00420-y

[8] Hanski I, von Hertzen L, Fyhrquist N, Environmental biodiversity, human microbiota, and allergy are interrelated. Proc Natl Acad Sci U S A. 2012;109(21):8334–8339. 22566627 10.1073/pnas.1205624109 PMC3361383

[9] van Dorst M, Pyuza JJ, Nkurunungi G, Immunological factors linked to geographical variation in vaccine responses. Nat Rev Immunol. 2024;24(4):250–263. 37770632 10.1038/s41577-023-00941-2

[10] Brodin P, Davis MM. Human immune system variation. Nat Rev Immunol. 2017;17(1):21–29. 27916977 10.1038/nri.2016.125 PMC5328245

[11] Kidd BA, Peters LA, Schadt EE, Dudley JT. Unifying immunology with informatics and multiscale biology. Nat Immunol. 2014;15(2):118–127. 24448569 10.1038/ni.2787 PMC4345400

[12] Davis MM, Tato CM, Furman D. Systems immunology: just getting started. Nat Immunol. 2017;18(7):725–732. 28632713 10.1038/ni.3768 PMC5790187

[13] Chen YH, Yeung F, Lacey KA, Rewilding of laboratory mice enhances granulopoiesis and immunity through intestinal fungal colonization. Sci Immunol. 2023;8(84):eadd6910. 37352372 10.1126/sciimmunol.add6910 PMC10350741

[14] Leung JM, Budischak SA, Chung The H, Rapid environmental effects on gut nematode susceptibility in rewilded mice. PLOS Biology. 2018;16(3):e2004108. 29518091 10.1371/journal.pbio.2004108 PMC5843147

[15] Chudnovskiy A, Mortha A, Kana V, Host-Protozoan Interactions Protect from Mucosal Infections through Activation of the Inflammasome. Cell. 2016;167(2):444–456 e414. 27716507 10.1016/j.cell.2016.08.076 PMC5129837

[16] Yeung F, Chen YH, Lin JD, Altered Immunity of Laboratory Mice in the Natural Environment Is Associated with Fungal Colonization. Cell Host Microbe. 2020;27(5):809–822 e806. 32209432 10.1016/j.chom.2020.02.015 PMC7276265

[17] Oyesola O, Downie AE, Howard N, Genetic and environmental interactions contribute to immune variation in rewilded mice. Nat Immunol. 2024;25(7):1270–1282. 38877178 10.1038/s41590-024-01862-5 PMC11224019

[18] Brodin P, Jojic V, Gao T, Variation in the human immune system is largely driven by non-heritable influences. Cell. 2015;160(1-2):37–47. 25594173 10.1016/j.cell.2014.12.020 PMC4302727

[19] Li Y, Oosting M, Smeekens SP, A Functional Genomics Approach to Understand Variation in Cytokine Production in Humans. Cell. 2016;167(4):1099–1110 e1014. 27814507 10.1016/j.cell.2016.10.017

[20] Rosshart SP, Herz J, Vassallo BG, Laboratory mice born to wild mice have natural microbiota and model human immune responses. Science. 2019;365(6452). 10.1126/science.aaw4361 PMC7377314 31371577

[21] Beura LK, Hamilton SE, Bi K, Normalizing the environment recapitulates adult human immune traits in laboratory mice. Nature. 2016;532(7600):512–516. 27096360 10.1038/nature17655 PMC4871315

[22] Chen YH, Zaldana K, Yeung F, Rewilding catalyzes maturation of the humoral immune system. Sci Adv. 2025;11(10):eads2364. 40053586 10.1126/sciadv.ads2364 PMC11887799

[23] Downie AE, Oyesola O, Barre RS, Spatiotemporal-social association predicts immunological similarity in rewilded mice. Sci Adv. 2023;9(51):eadh8310. 38134275 10.1126/sciadv.adh8310 PMC10745690

[24] Dill-McFarland KA, Tang ZZ, Kemis JH, Close social relationships correlate with human gut microbiota composition. Sci Rep. 2019;9(1):703. 30679677 10.1038/s41598-018-37298-9 PMC6345772

[25] Carr EJ, Dooley J, Garcia-Perez JE, The cellular composition of the human immune system is shaped by age and cohabitation. Nat Immunol. 2016;17(4):461–468. 26878114 10.1038/ni.3371 PMC4890679

[26] Rehermann B, Graham AL, Masopust D, Hamilton SE. Integrating natural commensals and pathogens into preclinical mouse models. Nat Rev Immunol. 2024. 10.1038/s41577-024-01108-3 PMC12126596 39562646

[27] Wagar LE, DiFazio RM, Davis MM. Advanced model systems and tools for basic and translational human immunology. Genome Med. 2018;10(1):73. 30266097 10.1186/s13073-018-0584-8 PMC6162943

[28] Estes JD, Wong SW, Brenchley JM. Nonhuman primate models of human viral infections. Nat Rev Immunol. 2018;18(6):390–404. 29556017 10.1038/s41577-018-0005-7 PMC5970954

[29] Flynn JK, Ortiz AM, Herbert R, Brenchley JM. Host Genetics and Environment Shape the Composition of the Gastrointestinal Microbiome in Nonhuman Primates. Microbiol Spectr. 2023;11(1):e0213922. 36475838 10.1128/spectrum.02139-22 PMC9927375

[30] Langner CA, Brenchley JM. FRugally Optimized DNA Octomer (FRODO) qPCR Measurement of HIV and SIV in Human and Nonhuman Primate Samples. Curr Protoc. 2021;1(4):e93. 33861500 10.1002/cpz1.93 PMC8054980

[31] Lin JD, Devlin JC, Yeung F, Rewilding Nod2 and Atg16l1 Mutant Mice Uncovers Genetic and Environmental Contributions to Microbial Responses and Immune Cell Composition. Cell Host Microbe. 2020;27(5):830–840 e834. 32209431 10.1016/j.chom.2020.03.001 PMC7228860

[32] Rosshart SP, Vassallo BG, Angeletti D, Wild Mouse Gut Microbiota Promotes Host Fitness and Improves Disease Resistance. Cell. 2017;171(5):1015–1028 e1013. 29056339 10.1016/j.cell.2017.09.016 PMC6887100

[33] Bacher P, Hohnstein T, Beerbaum E, Human Anti-fungal Th17 Immunity and Pathology Rely on Cross-Reactivity against Candida albicans. Cell. 2019;176(6):1340–1355 e1315. 30799037 10.1016/j.cell.2019.01.041

[34] Ueda Y, Yang K, Foster SJ, Kondo M, Kelsoe G. Inflammation controls B lymphopoiesis by regulating chemokine CXCL12 expression. J Exp Med. 2004;199(1):47–58. 14707114 10.1084/jem.20031104 PMC1887733

[35] Ueda Y, Kondo M, Kelsoe G. Inflammation and the reciprocal production of granulocytes and lymphocytes in bone marrow. J Exp Med. 2005;201(11):1771–1780. 15939792 10.1084/jem.20041419 PMC1952536

[36] Elhmouzi-Younes J, Palgen JL, Tchitchek N, In depth comparative phenotyping of blood innate myeloid leukocytes from healthy humans and macaques using mass cytometry. Cytometry A. 2017;91(10):969–982. 28444973 10.1002/cyto.a.23107

[37] Lin A, Liang F, Thompson EA, Rhesus Macaque Myeloid-Derived Suppressor Cells Demonstrate T Cell Inhibitory Functions and Are Transiently Increased after Vaccination. J Immunol. 2018;200(1):286–294. 29180488 10.4049/jimmunol.1701005

[38] Cain DW, Snowden PB, Sempowski GD, Kelsoe G. Inflammation triggers emergency granulopoiesis through a density-dependent feedback mechanism. PLoS One. 2011;6(5):e19957. 21655273 10.1371/journal.pone.0019957 PMC3104996

[39] Panopoulos AD, Watowich SS. Granulocyte colony-stimulating factor: molecular mechanisms of action during steady state and ‘emergency’ hematopoiesis. Cytokine. 2008;42(3):277–288. 18400509 10.1016/j.cyto.2008.03.002 PMC2852428

[40] Clapes T, Lefkopoulos S, Trompouki E. Stress and Non-Stress Roles of Inflammatory Signals during HSC Emergence and Maintenance. Front Immunol. 2016;7:487. 27872627 10.3389/fimmu.2016.00487 PMC5098161

[41] Baldridge MT, King KY, Goodell MA. Inflammatory signals regulate hematopoietic stem cells. Trends Immunol. 2011;32(2):57–65. 21233016 10.1016/j.it.2010.12.003 PMC3042730

[42] Ho NP, Takizawa H. Inflammation Regulates Haematopoietic Stem Cells and Their Niche. Int J Mol Sci. 2022;23(3). 10.3390/ijms23031125 PMC8835214 35163048

[43] Broxmeyer HE, Williams DE, Lu L, The suppressive influences of human tumor necrosis factors on bone marrow hematopoietic progenitor cells from normal donors and patients with leukemia: synergism of tumor necrosis factor and interferon-gamma. J Immunol. 1986;136(12):4487–4495. 3086433

[44] Selleri C, Sato T, Anderson S, Young NS, Maciejewski JP. Interferon-gamma and tumor necrosis factor-alpha suppress both early and late stages of hematopoiesis and induce programmed cell death. J Cell Physiol. 1995;165(3):538–546. 7593233 10.1002/jcp.1041650312

[45] Rezzoug F, Huang Y, Tanner MK, TNF-alpha is critical to facilitate hemopoietic stem cell engraftment and function. J Immunol. 2008;180(1):49–57. 18097003 10.4049/jimmunol.180.1.49

[46] Yamashita M, Passegue E. TNF-alpha Coordinates Hematopoietic Stem Cell Survival and Myeloid Regeneration. Cell Stem Cell. 2019;25(3):357–372 e357. 31230859 10.1016/j.stem.2019.05.019 PMC6733032

[47] Ito T, Hamazaki Y, Takaori-Kondo A, Minato N. Bone Marrow Endothelial Cells Induce Immature and Mature B Cell Egress in Response to Erythropoietin. Cell Struct Funct. 2017;42(2):149–157. 29070774 10.1247/csf.17018

[48] Giladi A, Paul F, Herzog Y, Single-cell characterization of haematopoietic progenitors and their trajectories in homeostasis and perturbed haematopoiesis. Nat Cell Biol. 2018;20(7):836–846. 29915358 10.1038/s41556-018-0121-4

[49] Bennett LF, Liao C, Quickel MD, Inflammation induces stress erythropoiesis through heme-dependent activation of SPI-C. Sci Signal. 2019;12(598). 10.1126/scisignal.aap7336 PMC6904953 31506384

[50] Millot S, Andrieu V, Letteron P, Erythropoietin stimulates spleen BMP4-dependent stress erythropoiesis and partially corrects anemia in a mouse model of generalized inflammation. Blood. 2010;116(26):6072–6081. 20844235 10.1182/blood-2010-04-281840

[51] Nairz M, Schroll A, Moschen AR, Erythropoietin contrastingly affects bacterial infection and experimental colitis by inhibiting nuclear factor-kappaB-inducible immune pathways. Immunity. 2011;34(1):61–74. 21256055 10.1016/j.immuni.2011.01.002 PMC3032045

[52] Paulson RF, Ruan B, Hao S, Chen Y. Stress Erythropoiesis is a Key Inflammatory Response. Cells. 2020;9(3). 10.3390/cells9030634 PMC7140438 32155728

[53] Mistry JJ, Marlein CR, Moore JA, ROS-mediated PI3K activation drives mitochondrial transfer from stromal cells to hematopoietic stem cells in response to infection. Proc Natl Acad Sci U S A. 2019;116(49):24610–24619. 31727843 10.1073/pnas.1913278116 PMC6900710

[54] Mistry JJ, Hellmich C, Moore JA, Free fatty-acid transport via CD36 drives beta-oxidation-mediated hematopoietic stem cell response to infection. Nat Commun. 2021;12(1):7130. 34880245 10.1038/s41467-021-27460-9 PMC8655073

[55] Zhang Y, Depond M, He L, CXCR4/CXCL12 axis counteracts hematopoietic stem cell exhaustion through selective protection against oxidative stress. Sci Rep. 2016;6:37827. 27886253 10.1038/srep37827 PMC5122894

[56] Zhu H, Kwak HJ, Liu P, Reactive Oxygen Species-Producing Myeloid Cells Act as a Bone Marrow Niche for Sterile Inflammation-Induced Reactive Granulopoiesis. J Immunol. 2017;198(7):2854–2864. 28235862 10.4049/jimmunol.1602006 PMC5360524

[57] Gruber JF, Hille DA, Liu GF, Heterogeneity of Rotavirus Vaccine Efficacy Among Infants in Developing Countries. Pediatr Infect Dis J. 2017;36(1):72–78. 27755463 10.1097/INF.0000000000001362

[58] French N, Nakiyingi J, Carpenter LM, 23-valent pneumococcal polysaccharide vaccine in HIV-1-infected Ugandan adults: double-blind, randomised and placebo controlled trial. Lancet. 2000;355(9221):2106–2111. 10902624 10.1016/s0140-6736(00)02377-1

[59] Kaul A, Schonmann U, Pohlmann S. Seroprevalence of viral infections in captive rhesus and cynomolgus macaques. Primate Biol. 2019;6(1):1–6. 32110713 10.5194/pb-6-1-2019 PMC7041514

[60] Ohtaki S, Kodama H, Hondo R, Kurata T. Activation of cytomegalovirus infection in immunosuppressed cynomolgus monkeys inoculated with varicella-zoster virus. Acta Pathol Jpn. 1986;36(10):1537–1552. 3026140 10.1111/j.1440-1827.1986.tb02825.x

[61] Itell HL, Kaur A, Deere JD, Barry PA, Permar SR. Rhesus monkeys for a nonhuman primate model of cytomegalovirus infections. Curr Opin Virol. 2017;25:126–133. 28888133 10.1016/j.coviro.2017.08.005 PMC5659282

[62] Simpson J, Starke CE, Ortiz AM, Multiple modes of antigen exposure induce clonotypically diverse epitope-specific CD8+ T cells across multiple tissues in nonhuman primates. Plos Pathogens. 2022;18(7):18. 10.1371/journal.ppat.1010611 PMC9262242 35797339

[63] Price DA, Brenchley JM, Ruff LE, Avidity for antigen shapes clonal dominance in CD8+ T cell populations specific for persistent DNA viruses. J Exp Med. 2005;202(10):1349–1361. 16287711 10.1084/jem.20051357 PMC2212993

[64] Council NR, Earth Do, Studies L, Research IfLA, Care CftUotGft, Animals UoL. Guide for the care and use of laboratory animals. 2010.

[65] Bar J, Leung JM, Hansen C, Strong effects of lab-to-field environmental transitions on the bacterial intestinal microbiota of Mus musculus are modulated by Trichuris murisinfection. FEMS Microbiol Ecol. 2020;96(10). 10.1093/femsec/fiaa167 32816007

[66] Ortiz AM, Flynn JK, DiNapoli SR, Antiretroviral Therapy Administration in Healthy Rhesus Macaques Is Associated with Transient Shifts in Intestinal Bacterial Diversity and Modest Immunological Perturbations. J Virol. 2019;93(18). 10.1128/JVI.00472-19 PMC6714794 31270225

