# Supplementary material for: Hematological Consequences of Environmental Change During Dewilding of Rhesus Macaques
Source: Res Sq. 2025 Oct 16:rs.3.rs-7767375. Preprint. [Version 1] doi: 10.21203/rs.3.rs-7767375/v1 (PMC12747276; doi:10.21203/rs.3.rs-7767375/v1)
Supplement: 1 [file NIHPPrs7767375v1-supplement-1.pdf]

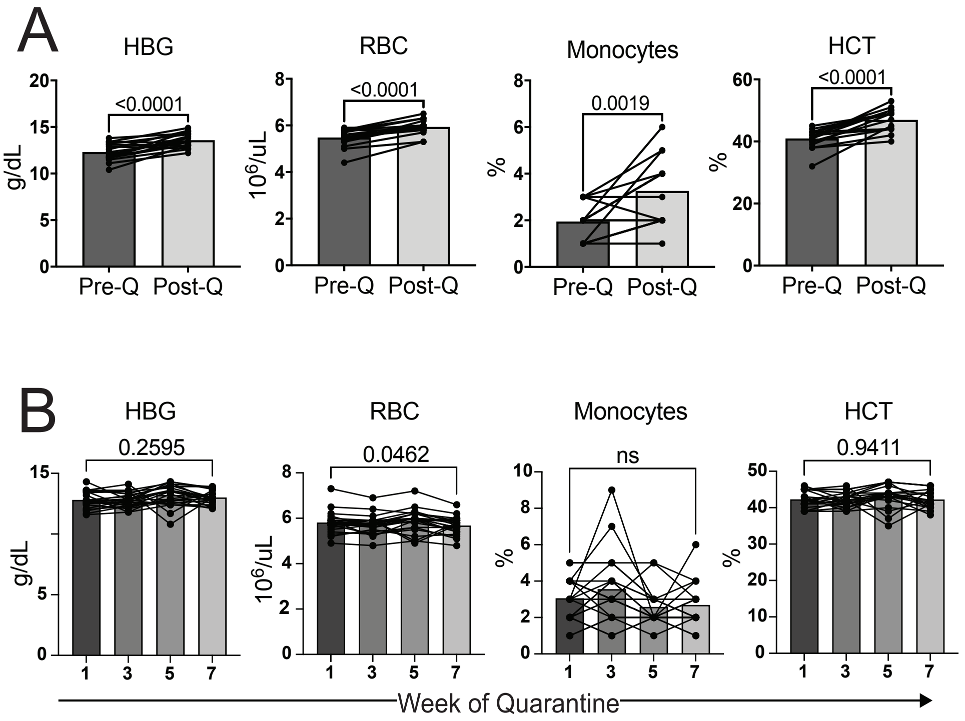

Supplemental Figure 1. Effect of dewilding on blood parameters. (A) Before-and-after bar plots showing the concentrations or percentages of hemoglobin (HBG), red blood cells (RBC), monocytes, and hematocrit (HCT) measured in rhesus macaques before (Pre-Q) and after 3 months of quarantine in a lab environment (Post-Q), as assessed by complete blood count (CBC) with differential (N=16). (B) Bar plots showing the concentration or percentages of hemoglobin (HBG), red blood cells (RBC), monocytes, and hematocrit (HCT) measured in rhesus macaques at weeks 1, 3, 5, and 7 of quarantine in a lab environment (Post-Q), as assessed by complete blood count (CBC) with differential (N=22). Dots in bar plots represent individual rhesus macaques. *P* values were determined by paired two-tailed t-test.

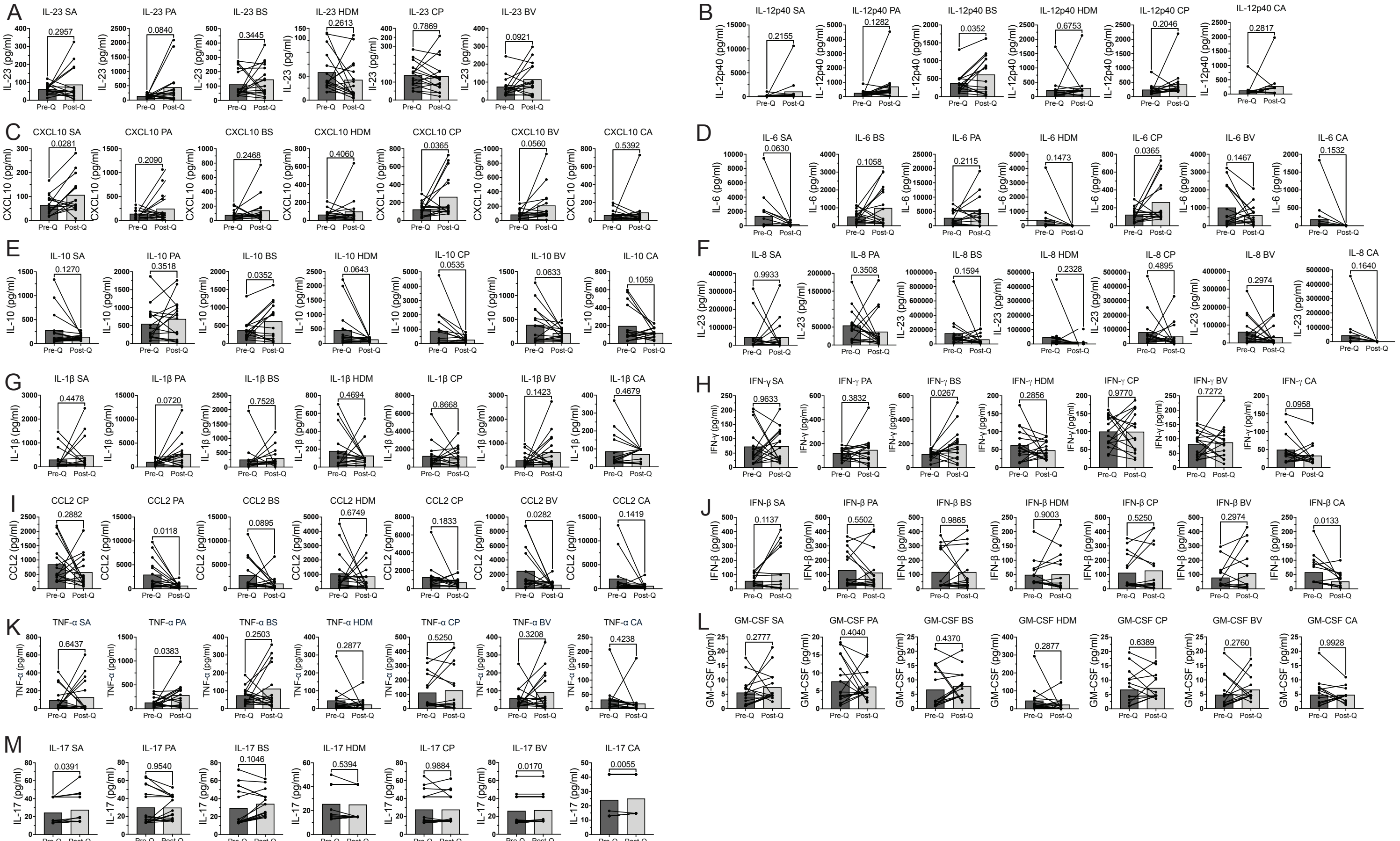

Supplemental Figure 2. Dewilding-driven shifts in pathogen-specific immune responses. (A) Before-and-after bar plots showing the cytokine response to in vitro stimulation by *Staphylococcus aureus* (SA), *Pseudomonas aeruginosa* (PA), *Bacillus subtilis* (BS), House Dust Mite (HDM), *Clostridium perfringens* (CP), and *Bacteroides vulgatus* (BV) as demonstrated by the concentration of IL-23, before and after quarantine (N=16). (B) Before-and-after bar plots showing the cytokine response to in vitro stimulation by *Staphylococcus aureus* (SA), *Pseudomonas aeruginosa* (PA), *Bacillus subtilis* (BS), House Dust Mite (HDM), *Clostridium perfringens* (CP), and *Candida albicans* (CA) as demonstrated by the concentration of IL-12p40, before and after quarantine (N=16). (C-M) Before-and-after bar plots showing the cytokine response to in vitro stimulation by *Staphylococcus aureus* (SA), *Pseudomonas aeruginosa* (PA), *Bacillus subtilis* (BS), House Dust Mite (HDM), *Clostridium perfringens* (CP), *Bacteroides vulgatus* (BV) and *Candida albicans* (CA) as demonstrated by the concentration of CXCL10, IL-6, IL-10, IL-8, IL-1β, IFN-γ, CCL2, IFN-β, TNF-α, GM-CSF, and IL-17, respectively, before and after quarantine (N=16). Dots in bar plots represent individual rhesus macaques. *P* values were determined by paired two-tailed t-test.

A. Chemokine Panel

	Laser	Target	Clone
1	PE/Fire810	CD197 (CCR7)	G043H7
2	BV480	CD183 (CXCR3)	1C6/CXCR3
3	PerCP-eFluor710	CD185 (CXCR5)	MU5UBEE

B. Intracellular Panel

	Laser	Target	Clone
1	AF532	IL-2	MQ1-17H12
2	AF660	TNFa	MAb11
3	APC	IL-5	JES1-39D10
4	BV421	IL-13	JES10-5A2
5	eFluor450	IL-17	eBio64DEC17
6	AF647	Foxp3	150D
	AF555	Ki-67	B56
8	BV510	IFN-g	B27
9	R718	Granzyme B	GB11
10	AF532	IL-2	MQ1-17H12
11	AF660	TNFa	MAb11
12	APC	IL-5	JES1-39D10
13	BV421	IL-13	JES10-5A2
14	eFluor450	IL-17	eBio64DEC17
15	AF647	Foxp3	150D
16	AF555	Ki-67	B56
17	BV510	IFN-g	B27
18	R718	Granzyme B	GB11

C. Surface Receptor Panel

	Laser	Target	Clone
1	BUV395	CD3	SP34.2
2	BB700	CD4	OKT4
3	APC/Fire810	CD8	SK1
4	BV605	CD45RA	5HP
5	BV785	CD279 (PD-1)	EH12.2H7
6	PE-Cy5	CD278 (ICOS)	C398.4A
	BUV496	CD16	3G8
8	BUV563	CD14	M5E2
9	BUV737	CD56	NCAM16.2
10	BUV805	CD20	2H7
11	PE-FIRE640	CD69	FN50
12	BV650	CD28	CD28.2
13	BV711	HLA-DR	L243
14	BV750	CD27	O323
15	BB515	CD95	DX2
16	PerCP-Cy5.5	CD38	OKT10
17	PE	CD159a	Beckman Coulter
18	PE/Dazzle 594	CD11b	ICRF44
19	PE-Alexa 700	CD25	LIFE TECH
20	PE-Cy7	TCRgd	B1
21	APC-Cy7	CD314 (NKG2D)	1D11
22	BUV661	CD86	2331 (FUN-1)

Supplemental Figure 3. Panel design for flow cytometry of peripheral blood mononuclear cells.

Panel designs for flow cytometry of chemokines (A), intracellular markers (B), and surface receptors (C) in of peripheral blood mononuclear cells (PBMCs).

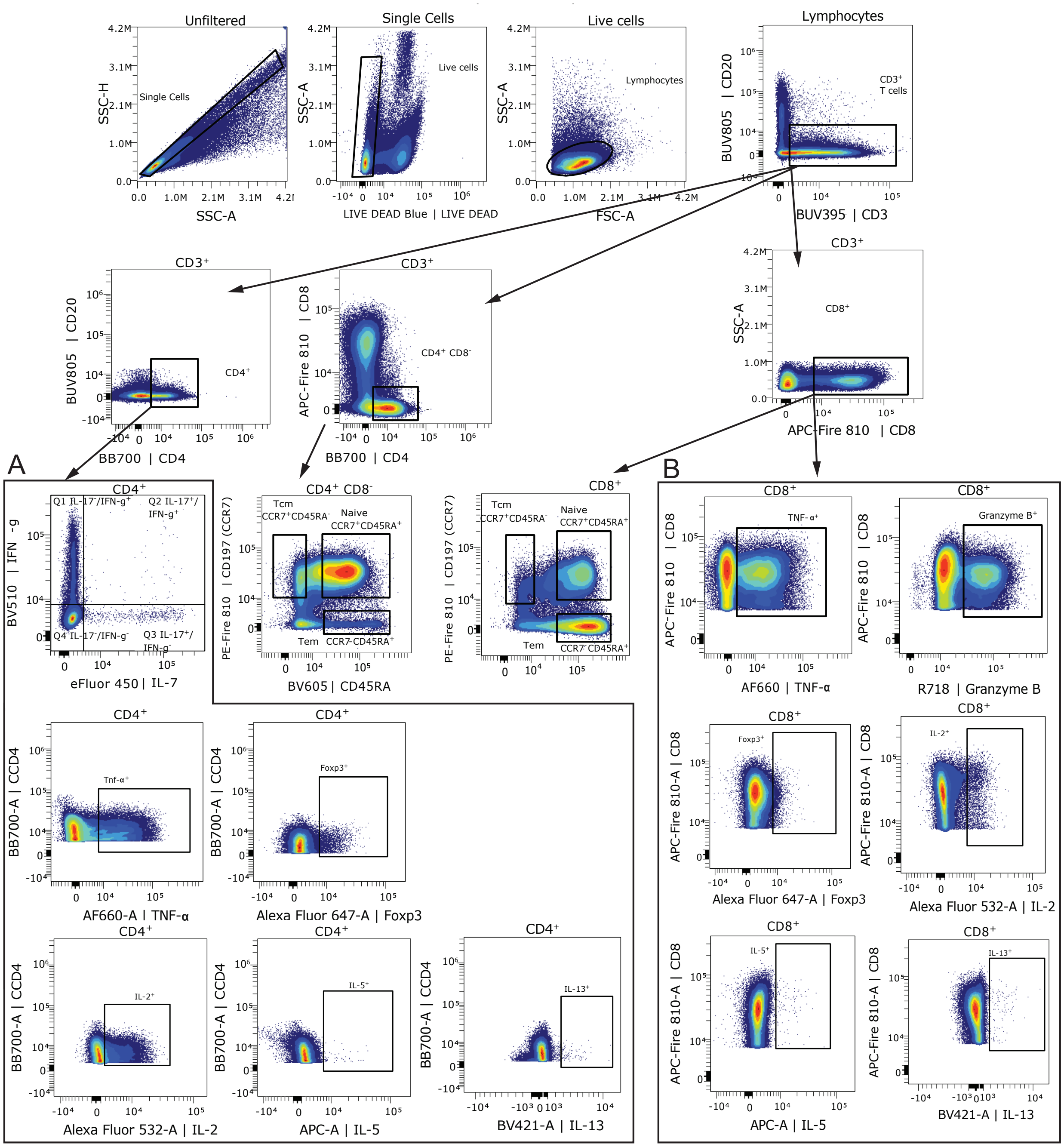

Supplemental Figure 4. Gating strategy for peripheral blood mononuclear cell T cell flow cytometry. $CD4^+$ and $CD8^+$ populations were gated by gating on lymphocytes, singlets and live cells that were either $CD4^+CD20^-$ (A), $CD4^+CD8^-$, or $CD8^+$. $CD4^+CD8^-$ and $CD8^+$ populations were gated for Central Memory ($CCR7^+$ $CD45RA^-$), Effector Memory ($CCR7^-$CD45RA-) and Naïve T cells ($CCR7^+CD45RA^+$). (A) $CD4^+CD20^-$ was gated for IFN-g, IL-17, TNF- α, Foxp3, IL-2, IL-5, and IL-13. (B) $CD8^+$ was gated for TNF- α, Granzyme-B, Foxp3, IL-2, IL-5, and IL-13.

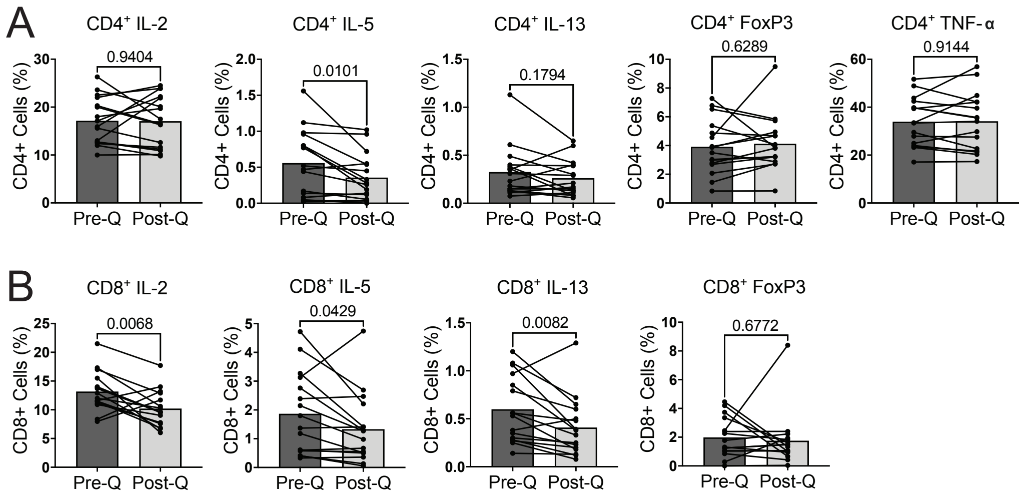

Supplemental Figure 5. Dewilding-driven shifts in T-cell cytokine production. (A) Before-and-after bar plots showing the intracellular cytokine staining for IL-2, IL-5, IL-13, FoxP3, and TNF-α in CD4+ T cells, before and after quarantine (N=16). (B) Before-and-after bar plots showing the intracellular cytokine staining for IL-2, IL-5, IL-13, and FoxP3 in CD8+ T cells, before and after quarantine (N=16). Dots in bar plots represent individual rhesus macaques. *P* values were determined by paired two-tailed t-test.

A. Hematopoietic Stem and Progenitor Cell Panel

	Laser	Target	Clone	Company	Antibody Catalog #	Dilution Factor
1	BUV395	CD45RA	5H9	BD Horizon	569489	200
2	BUV563	CD14	M5E2	BD OptiBuild	741441	400
3	BUV737	CD56	NCAM16.2	BD Horizon	612766	400
4	BUV805	CD20	2H7	BD Horizon	612905	400
5	BV421	CD90	5E.10	BD Horizon	562556	25
6	V450	CD45	D058-1283	BD Horizon	561291	25
7	BV650	CD34	563	BD OptiBuild	740605	100
8	FITC	CD123	7G3	BD Pharmingen	558663	100
9	PE	CD66	TET2	Miltenyi Biotec	130-177-699	400
10	PE-CF 594	CD3	SP34-2	BD Horizon	562406	400
11	PE-Cy5	CD10	HI10a	BD Pharmingen	555376	200
12	PE-Cy7	CD117 (cKit)	104D2	BioLegend	313211	400
13	APC-Cy7	CD38	OKT10	CapricoBio	100895	200
		Fc Block	Human TruStain	BioLegend	422302	100

B. Neutrophil Progenitor and $CD34^{-}$ Panel

	Laser	Target	Clone	Company	Antibody Catalog #	Dilution Factor
1	BUV395	CD3	SP34-2	BD Horizon	564117	400
2	BUV496	CD16	3G8	BD Horizon	612944	400
3	BUV563	CD14	M5E2	BD OptiBuild	741441	400
4	BUV737	CD56	NCAM16.2	BD Horizon	612766	400
5	BUV805	CD20	2H7	BD Horizon	612905	400
6	V450	CD45	D058-1283	BD Horizon	561291	25
	BV510	CD11b	ICRF44	BioLegend	301334	200
8	BV650	CD34	563	BD OptiBuild	740605	100
9	BV711	HLA-DR	L243	BioLegend	307644	200
10	BV785	CD11c	3.9	BioLegend	301644	200
11	FITC	CD123	7G3	BD Pharmingen	558663	100
12	BB700	CD4	OKT4	BD Horizon	566829	100
13	PE	CD66	TET2	Miltenyi Biotec	130-177-699	400
14	PE-Cy5	CD10	HI10a	BD Pharmingen	555376	200
15	RB780	CD32	FLI8.26	BD OptiBuild	756003	100
16	APC	CD87	VIM5	Miltenyi Biotec	130-099-456	100
17	APC-Vio770	CD33	AC104.3E3	Miltenyi Biotec	130-113-346	400
18	APC/Fire 810	CD8	SK1	BioLegend	344764	100
		Fc Block	Human TruStain	BioLegend	422302	100

Supplemental Figure 6. Panel design for flow cytometry of bone marrow. Panel designs for flow cytometry of hematopoietic stem and progenitor cells (A) and neutrophil progenitors and other $CD34^{-}$ immune cells (B).

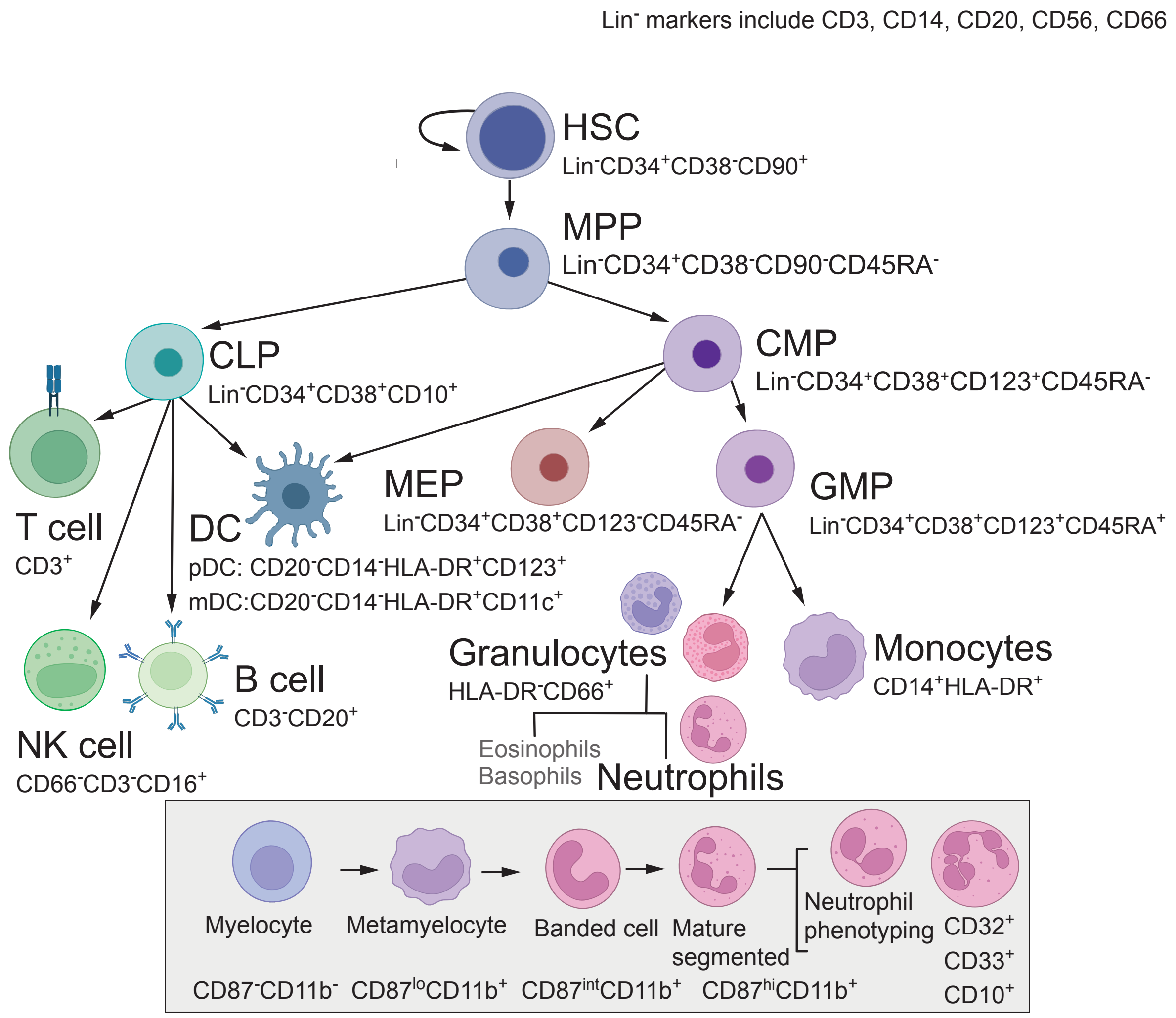

Supplemental Figure 7. Bone marrow immune cell population lineage assessed by flow cytometry.

Schematic depicting lineages and maturation of the immune cell populations assessed by flow cytometry of rhesus macaque bone marrow samples. Immune cell populations include hematopoeitc stem cells (HSC), multipotent progenitor (MPP) cells, common lymphoid progenitor (CLP) cells, megakaryocyte-erythrocyte progenitor (MEP) cells, common myeloid progenitor (CMP) cells, and granulocyte-monocyte progenitor (GMP) cells. Additionally, T cells, dendritic cells (DC), natural killer (NK) cells, B cells, granulocytes, and monocytes were assessed. The maturation stages of neutrophils assessed were myelocytes, metamyelocytes, banded neutrophils, and mature segmented neutrophils.

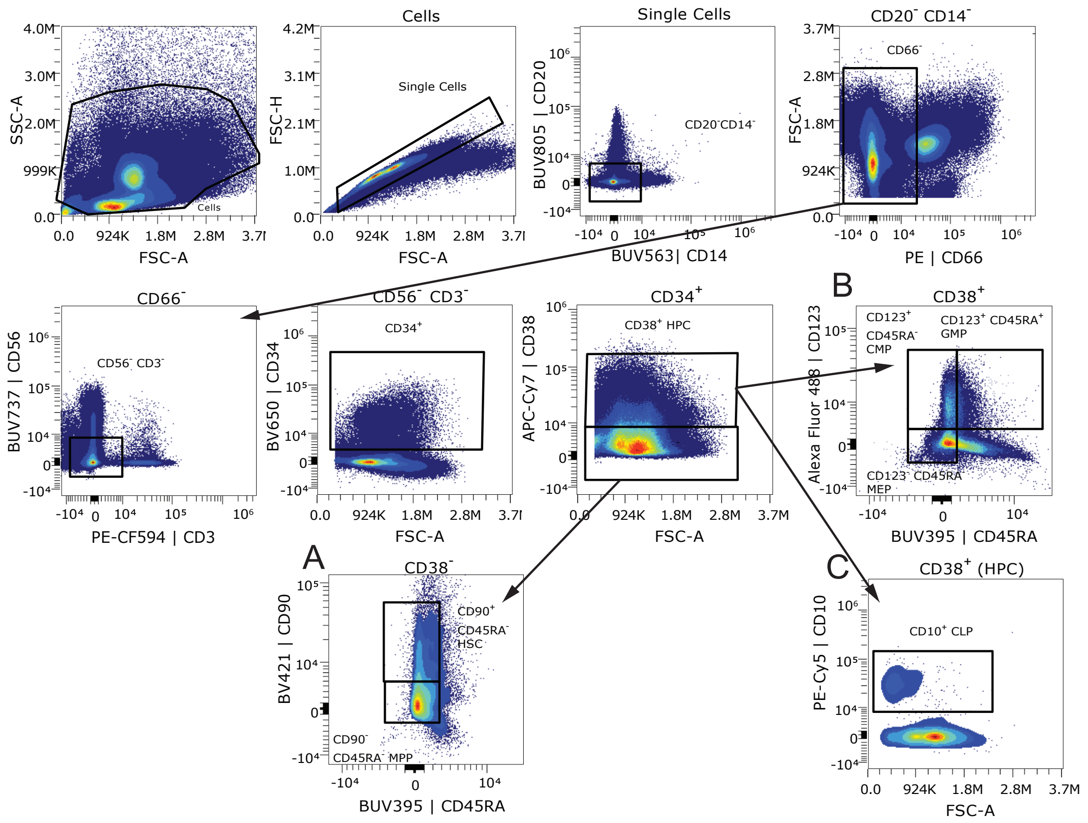

Supplemental Figure 8. Gating strategy for bone marrow hematopoietic stem and progenitor cell flow cytometry. The CD34+ population (HSC compartment) was gated by selecting cells, singlets, and excluding lineage markers (CD20- CD14- CD66- CD56- CD3-). Hematopoietic progenitor cells (HPC) were identified within the CD34+ population by gating on CD38+. (A) From the CD38+ HPC population, common myeloid progenitors (CMP) were identified as CD123+ CD45RA-, granulocyte-monocyte progenitors (GMP) as CD123+ CD45RA+, and megakaryocyte-erythroid progenitors (MEP) as CD123- CD45RA-. (B) From the CD38+ HPC population, common lymphoid progenitors (CLP) were identified as CD10+. (C) From the CD38- population, the least mature hematopoietic stem cells (HSC) were identified as CD90+ CD45RA-, and multipotent progenitor cells (MPP) as CD90- CD45RA-.

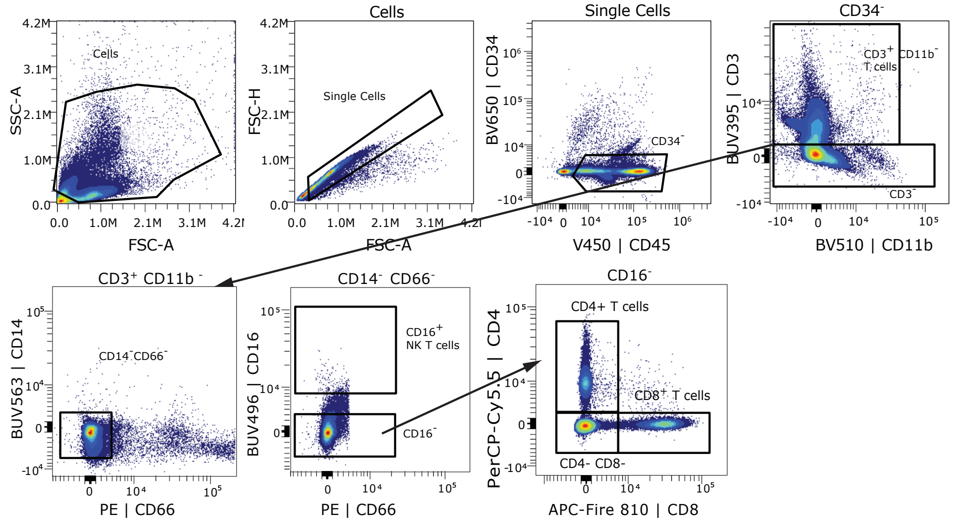

Supplemental Figure 9. Flow cytometry gating strategy for bone marrow T cells. The CD34- population (non-HSC) was gated by selecting cells and singlets. T cells were identified within the CD34- population by gating on CD3+ CD11b-. From the CD3+ CD11b- population, monocytes and granulocytes were excluded using CD14- CD66-, followed by gating for NK T cells with CD16+. Subsequently, CD4+ and CD8+ T cells were gated from the CD16- population.

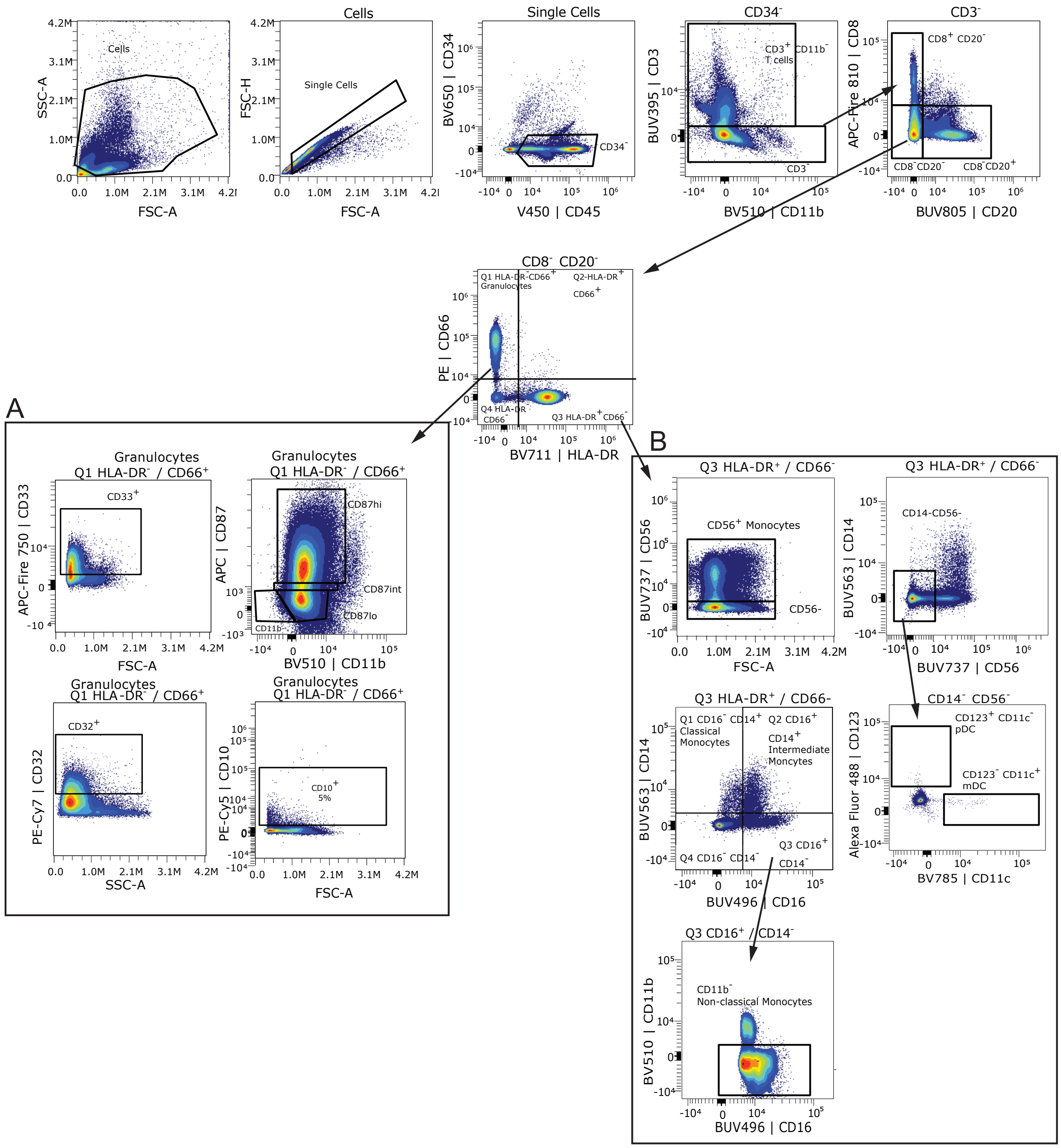

Supplemental Figure 10. Flow cytometry gating strategy for bone marrow granulocytes and myeloid cell populations. The CD34- population (non-HSC) was gated by selecting cells and singlets. Within the CD34- population, we excluded CD3+ T cells. (A) From the CD3- population, B cells were excluded using CD8-CD20-, and granulocytes were identified by gating on HLA-DR- CD66+ (Q1). Neutrophils were further phenotyped based on CD87 and CD11b expression, identifying CD11b+CD87lo metamyelocytes, CD11b+CD87int banded neutrophils, and CD11b+CD87hi mature segmented neutrophils. Additional neutrophil phenotyping was

performed using CD32, CD33, and CD10 expression. (B) Myeloid cell populations were gated from the CD3- population by excluding B cells (CD8-CD20-) and selecting HLA-DR+ CD66- (Q3). Monocyte subsets were assessed as CD56+ monocytes, classical monocytes (Q1: CD16-CD14+), intermediate monocytes (Q2: CD16- CD14-), and nonclassical monocytes (Q3: CD16+ CD14-, CD11b-). From the Q3 HLA-DR+ CD66- population, plasmacytoid dendritic cells (pDCs) were identified as CD123+ CD11c-, and myeloid dendritic cells (mDCs) as CD123- CD11c+.

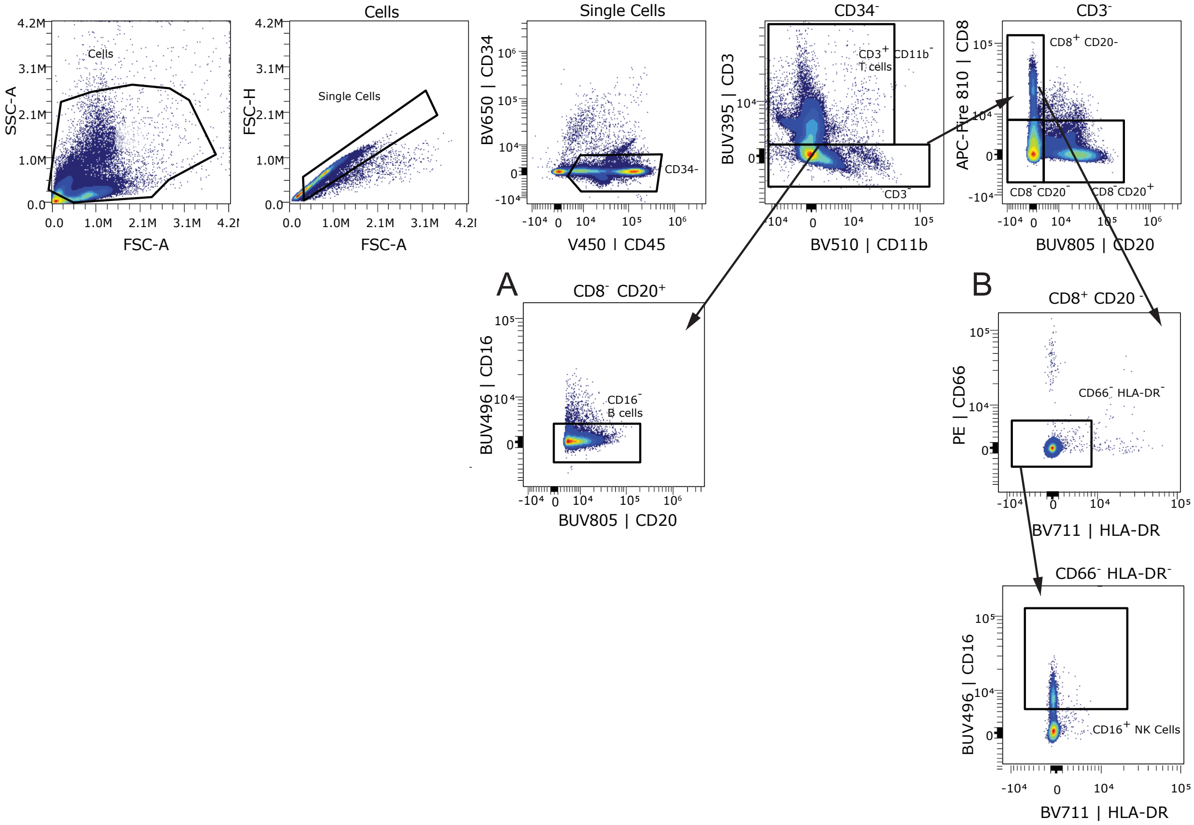

Supplemental Figure 11. Flow cytometry gating strategy for bone marrow B cells and natural killer cells. The CD34- population (non-HSC) was gated by selecting cells and singlets. Within the CD34- population, we excluded CD3+ T cells. (A) B cells were gated from the CD3- population using CD8- CD20+ and CD16-. (B)Natural killer (NK) cells were identified from the CD3- population by gating on CD8+CD20-, CD66- HLA-DR-, and CD16+.

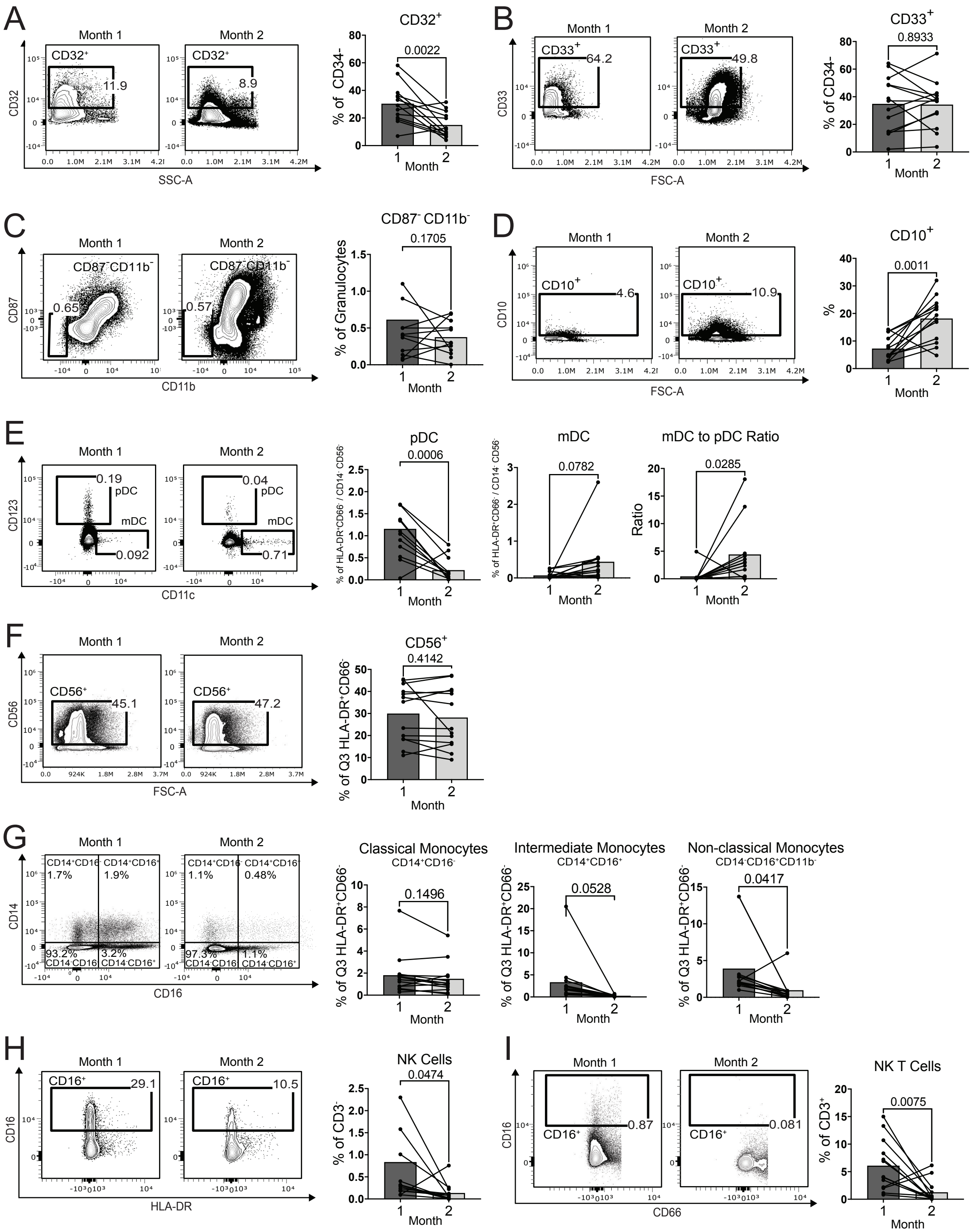

Supplemental Figure 12. Flow cytometry for bone marrow CD34- cell populations. (A) Representative flow

cytometry analysis and quantification of $CD32^+$ granulocytes at month 1 and month 2 of dewilding (N=13). (B) Representative flow cytometry analysis and quantification of $CD33^+$ granulocytes at month 1 and month 2 of dewilding (N=13). (C) Representative flow cytometry analysis and quantification of $CD87^-CD11b^-$ granulocytes at month 1 and month 2 of dewilding (N=13). (D) Representative flow cytometry analysis and quantification of $CD10^+$ granulocytes at month 1 and month 2 of dewilding (N=13). (E) Representative flow cytometry analysis and quantification of $CD123^+CD11c^-$ pDC and $CD123^-CD11c^+$ mDC at month 1 and month 2 of dewilding, along with quantification of the mDC to pDC ratio (N=13). (F) Representative flow cytometry analysis and quantification of $CD56^+$HLA-DR^-CD66^- monocytes at month 1 and month 2 of dewilding (N=13). (G) Representative flow cytometry analysis and quantification of $CD14^-CD16^-$ classical monocytes, $CD14^+CD16^+$ intermediate monocytes, and $CD14^-$ $CD16^+CD11b^-$ non-classical monocytes at month 1 and month 2 of dewilding (N=13). (H) Representative flow cytometry analysis and quantification of $CD16^+CD3^-$ natural killer (NK) cells at month 1 and month 2 of dewilding (N=13). (I) Representative flow cytometry analysis and quantification of $CD16^+CD3^+$ natural killer (NK) T cells at month 1 and month 2 of dewilding (N=13). Dots in bar plots represent individual rhesus macaques. *P* values were determined by paired two-tailed t-test.

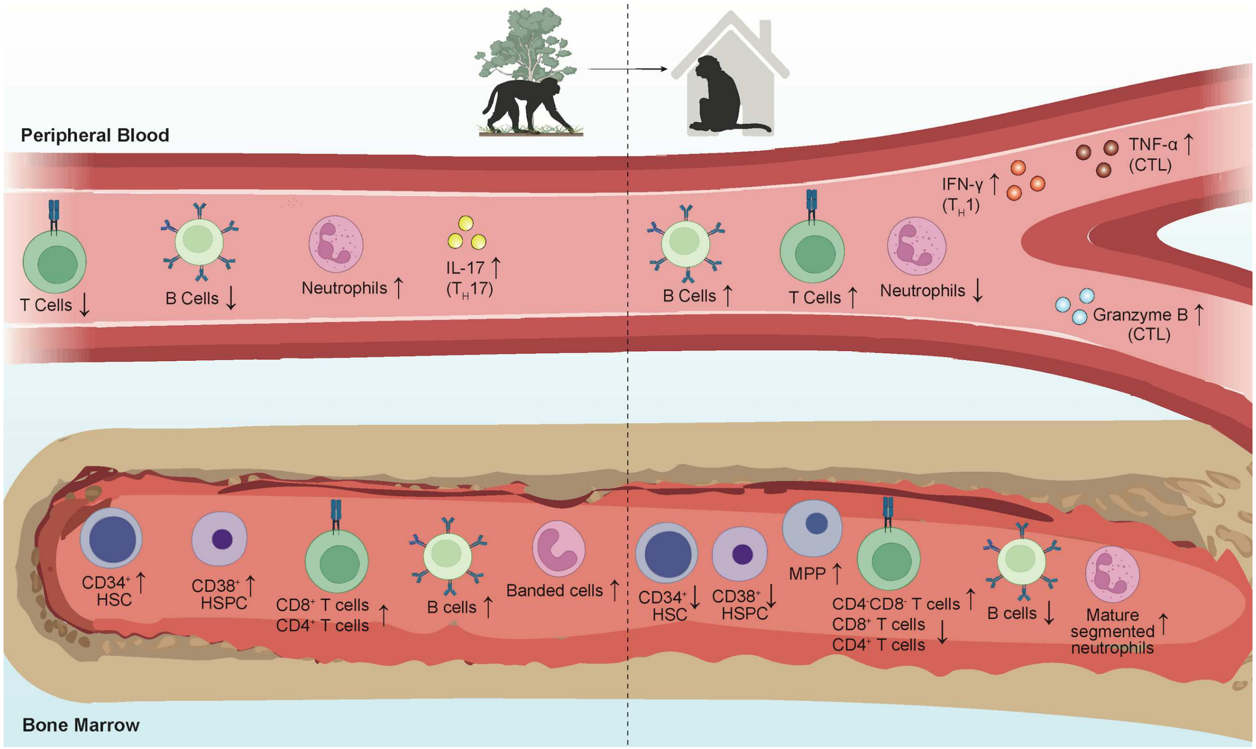

Supplemental Figure 13. Summary of dewilding-induced immune cell changes in peripheral blood and bone marrow. Effects of dewilding in a rhesus macaque model investigating changes in peripheral blood and bone marrow. Dewilding induced alterations in immune cell populations and their immune responses are depicted as cells with their respective trends (increase or decrease), shown on either side of the dashed line representing the dewilding process.
